# Just a phase? Mapping the transition of behavioural problems from childhood to adolescence

**DOI:** 10.1007/s00127-020-02014-4

**Published:** 2021-02-11

**Authors:** Joe Bathelt, Anna Vignoles, Duncan E. Astle

**Affiliations:** 1 Department of Psychology, Royal Holloway, University of London, TW20 0EX Egham, Surrey, UK; 2 Faculty of Education, University of Cambridge, Cambridge, UK; 3 MRC Cognition and Brain Sciences Unit, University of Cambridge, Cambridge, UK

**Keywords:** Adolescence, Childhood, Development, Education, Mental health, Nosology

## Abstract

**Purpose:**

Young people change substantially between childhood and adolescence. Yet, the current description of behavioural problems does not incorporate any reference to the developmental context. In the current analysis, we aimed to identify common transitions of behavioural problems between childhood and adolescence.

**Method:**

We followed 6744 individuals over 6 years as they transitioned from childhood (age 10) into adolescence (age 16). At each stage, we used a data-driven hierarchical clustering method to identify common profiles of behavioural problems, map transitions between profiles and identify factors that predict specific transitions.

**Results:**

Common profiles of behavioural problems matched known comorbidity patterns but crucially showed that the presentation of behavioural problems changes markedly between childhood and adolescence. While problems with hyperactivity/impulsivity, motor control and conduct were prominent in childhood, adolescents showed profiles of problems related to emotional control, anxiety and inattention. Transitions were associated with socio-economic status and cognitive performance in childhood

**Conclusion:**

We show that understanding behavioural difficulties and mental ill-health must take into account the developmental context in which the problems occur, and we establish key risk factors for specific negative transitions as children become adolescents.

**Supplementary Information:**

The online version contains supplementary material available at 10.1007/s00127-020-02014-4.

## Introduction

Multiple theoretical perspectives agree that psychopathology is a neurodevelopmental process starting early in life, or even before birth, that evolves across the lifespan [33, 53]. So, whilst adolescence is the most common period for the first onset of recognisable mental health difficulties [35], behavioural problems faced by adolescents likely have their roots in earlier symptoms or mechanisms. Despite this, current approaches to characterising psychopathology are mostly static with little reference to the developmental context in which the problems occur [48]. This presents a major challenge to the identification and treatment of these problems. Consequently, the approach to treating adolescent behavioural difficulties is largely reactionary, i.e. problems are only treated once they escalate. This is believed to be a major contributing factor to healthcare systems being overwhelmed by the demand to treat mental health difficulties and associated conditions [10, 15]. Therefore, healthcare systems worldwide have ambitions to move towards a proactive model of support and prevention [25]. But this ambition is dependent upon understanding how behavioural problems manifest at different points in development, how children transition into adolescence, and which children are most vulnerable to negative transitions like mental health difficulties and their consequences on educational attainment and wellbeing [17]. The purpose of this study is to provide this evidence base using a population-representative national birth cohort, thereby, improving the description of behavioural difficulty trajectories over child and adolescent development.

The transition between childhood and adolescence is linked to particularly striking changes in behaviour [18]. Adolescence is also a period of pronounced physical change associated with puberty, including sexual maturation and gain in weight, height and physical strength. There are also increasingly well-documented changes in brain structure and function, particularly in the maturation of the frontal lobe [6]. In addition to physical changes, adolescence is accompanied by increases in mental capabilities [16, 18], particularly in reasoning, decision-making and planning. In addition to marked changes within an individual, adolescence sees large changes in social and societal context, both of which may affect behaviour and behavioural problems. Adolescents are expected to take up more adult societal roles in most cultures [18] and personal relationships change, with a transition from parents as the most significant source of emotional support to same-sex peers [27].

Given these changes, one may expect a difference in the most prevalent behavioural problems in adolescence [14]. Indeed, some psychiatric and behavioural disorders that are highly prevalent in childhood are less common in adolescence and vice versa. For instance, only 40% of children diagnosed with ADHD meet diagnostic criteria in adolescence [42]. This may reflect a true difference in behavioural patterns between childhood and adolescence or may be tied to the particular diagnostic criteria. Diagnostic categories are defined through a list of behaviours that make no specific reference to developmental stage, beyond broad statements about age-appropriate behaviour. For example, for a DSM-V diagnosis of Attention Deficit Hyperactivity Disorder (ADHD), 6 out of 18 behaviours associated with inattention or hyperactivity/impulsivity must be present that are “inconsistent with the developmental level” [3]. Indeed, the omission of more specific criteria relating to development has been identified as a major shortcoming by the DSM-V workgroup [48]. Alternative conceptualisations that provide a better fit with psychometric data suggest that psychopathology is better understood as a combination of higher level dimensions, e.g. internalising–externalising, internalising–negative emotionality fear [36, 37, 59]. While dimensional models provide a better account of the data, they have yet to be translated directly into clinical practice, where treatment and support decisions need to be made that require a clear demarcation of “cases” [26, 61]. Recent efforts have employed methods from machine learning to combine good data fit with a clear stratification of individuals based on objective criteria. In contrast to traditional diagnostic systems like the International Classification of Diseases (ICD) or the DSM, these data-driven approaches identify an optimal subdivision using the data themselves, resulting in maximum homogeneity within each group and minimum overlap between the groups. Studies using these methods show that subgroups with distinct behavioural or cognitive profiles exist and are closely tied to potential neurobiological mechanisms [4, 5, 23, 32, 40, 57].

In the current study, we used a data-driven clustering method to identify subgroups of behavioural problem profiles in a sample of 6744 individuals taken from the British Cohort Study [7]—a prospective longitudinal study that followed all children born in England, Scotland and Wales born within the same week in April 1970 as they grew up. Following longstanding conceptualisations about the hierarchical structure of behavioural problems across development [2, 36], we based the analysis on a hierarchical clustering method. The current analysis focused on the transition between childhood and adolescence when participants were 10 and 16 years old. We identified consistent profiles of behavioural problems and their association with demographic variables, academic attainment and cognitive ability in childhood and adolescence. We then characterised transitions between childhood and adolescence to identify factors that predict transitions in behavioural problems.

## Methods

### Participants

The analysis is based on the 1970-British Cohort Study [7, 21]. The study followed the lives of 16,571 children who were born in the same week in 1970 in England, Scotland, Wales and Northern Ireland. The focus of the first survey was on the circumstances and outcomes of birth. Over the years, the study broadened its focus to cover many aspects of cohort members and their families such as health, education, employment and social development. Nine sweeps have been collected at the time of writing with another sweep planned for 2020. This cohort is ideal for an analysis like this because of its size, the careful sampling that aimed for population-representativeness^1^ and the well-timed data collection windows. Furthermore, this study is still ongoing, meaning that in the future, our findings can be related to longer term health, educational and occupational outcomes, as individuals’ transition into older age. Cohorts of this size, longevity, representativeness, scope and richness are rare. For the current analysis, childhood data ([7]; 1970, Sweep 2, 10-year-olds study, *n* = 14,874, https://doi.org/10.5255/UKDA-SN-3723-7) and adolescence data (1986, Sweep 3, 16-year-olds, *n* = 11,621, https://doi.org/10.5255/UKDA-SN-3535-4) were used. The sixth edition of the data (version of June 2016) was accessed online from the Centre for Longitudinal Studies (see Centre for Longitudinal Studies: http://www.cls.ioe.ac.uk). See Table 1 for demographic characteristics of the childhood and adolescent samples. The collection of these data predated the establishment of centralised Ethics review boards in the United Kingdom. Therefore, no information about Ethical review is available for the initial sweeps of the BCS.

**Table 1:** Demographic characteristics of the childhood and adolescent sample. Please note that the adolescent sample is a subsample of the childhood sample. The proportions are, therefore, not independent. For factors associated with attrition in the BCS1970 cohort, please see Ketende, McDonald, & Dex [34]

	Childhood		Adolescence	
BCS70 Sweep	Sweep 2		Sweep 3	
*n* (included)	12,134		6744	
Age [months]	117.75*	± 3.596	186.21	± 4.088
Female	48.87	5930	51.44	3469
Country of birth				
England	81.37	9874	83.76	5649
Scotland	10.06	1221	9.15	617
Wales	5.50	667	5.25	354
Other country	1.99	242	1.39	94
Northern Ireland	0.22	27	0.24	16
Republic of Ireland	0.07	9	0.06	4
Ethnicity				
English	94.59	11,478	96.59	6,514
Indian	1.41	171	1.44	97
Mixed/other	0.35	43	0.33	22
Pakistani	0.76	92	0.79	53
West Indian	1.10	133	1.08	73
Other European	0.54	66	0.52	35
Irish	0.48	58	0.65	44
Bangladeshi	0.07	8	0.06	4

Values are mean ± SD or % (*n*)

^*^Calcuated as the difference between the date of birth and the completion of the Maternal Self-Completion Form

### Ratings of behavioural problems

Both the childhood and adolescence survey included a parent questionnaire with questions relating to behavioural difficulties (Maternal Self-Completion Form MSF). The behavioural questionnaire consisted of a combination of items from “Rutter—A scale of behavioural deviance” Rutter et al. [53]) and the Hyperactivity/Behavioural Scale Conners et al. [12], see Supplementary Materials for a full list of items). For the childhood survey, the behavioural questions were rated on visual analogue scales ranging from “Does not apply” to “Certainly applies” (length: 100 mm). The distance between the starting point and the marker placed by the participant was contained in the BCS data. For the adolescence sweep, discrete scales with 4 points were used. We converted the childhood ratings to a 3- and 4-point discrete scales using kernel density estimation (KDE) to make the data comparable between the two time points. KDE is a method to create smooth distributions from data points that can then be divided into intervals.

Teacher ratings of behavioural problems were also obtained in childhood but only covered around a third of the sample in adolescence due to a teacher strike in 1986. Because of the missing data, the longitudinal associations between teacher ratings could not be analysed and we, therefore, opted to not include the teacher ratings in the current analysis.

**Fig. 1:**
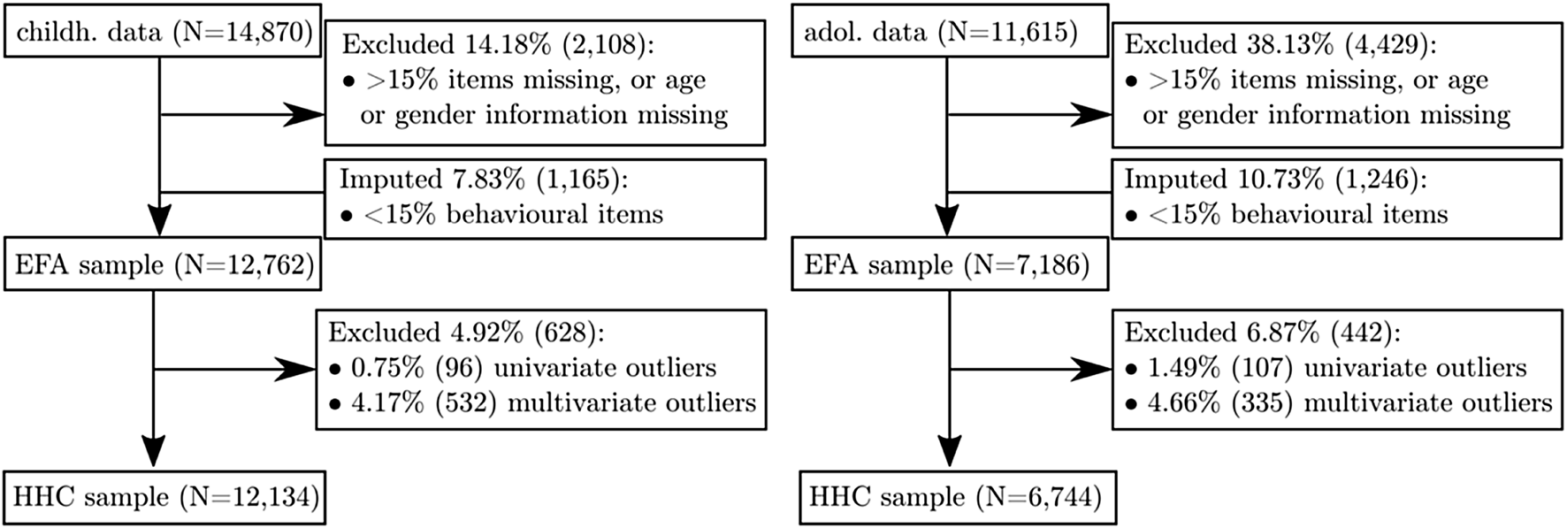
CONSORT diagram of data exclusion for the childhood and adolescence survey. *EFA* exploratory factor analysis, HHC hybrid hierarchical clustering

All participants in the childhood and adolescence data with complete ratings on all behavioural items were initially entered in the current analysis. The childhood data included parental ratings of behavioural problems for 14,870 children and the adolescence data included ratings for 11,615 young people. We excluded cases that were missing information about the exact age, i.e. date the questionnaire was completed, or the gender of the child. Further, cases with an excessive number of missing items were excluded. We used a cut-off of > 5 missing items (~ 15%). In the majority of cases above this cut-off, most questionnaire items were missing. Values were imputed for cases with fewer missing items using the expectation maximisation algorithm, which preserves the means, variances and covariance of the data and leads to less biased standard error estimates compared to mean or regression substitution Dempster et al. [19]. Questionnaire measures may show extreme responses that are not related to the content of the questionnaire that arises due to unintended, extreme, fake, or random responses. To account for those cases, we removed univariate, defined as a score more than three standard deviations below or above the mean across the sample, and multivariate outliers, defined as Mahalanobis distance above the recommended cut-off for the given number of dimensions. This procedure provides an effective way to remove extreme or unreliable scores [64]. After removal of cases with excessive missing data and removal of outliers, 12,134 children and 6684 adolescents were used for hybrid hierarchical clustering (see Fig. 1 for a breakdown of participants included at each stage of the analysis).

### Assessments of cognition, educational attainment and mental health

Direct assessments of cognitive performance, educational attainment and mental health were obtained at both time points. A full description of the assessments is available from the Centre for Longitudinal Studies (childhood: http://doi.org/10.5255/UKDA-SN-3723-7, adolescence: http://doi.org/10.5255/UKDA-SN-3535-4). In the childhood data, the cognitive assessment for children consisted of two verbal (word definitions, similarities) and two non-verbal tests (matrix reasoning, digit recall) from the British Ability Scales. For the adolescent assessment, the cognitive assessment consisted of the matrix reasoning subtest of the British Ability Scales. Educational assessments covered reading and mathematics (see Supplement for a detailed description). For all assessments, subtests were summarised per domain as one total score and raw scores were scaled to a mean of 0 and unit variance by subtracting the mean across the sample and dividing by the standard deviation (*z*-score). To compare differences in mental health, the rating of “emotional or behavioural problems” on the Medical Examination Form was used, which was filled in by a community medical officer or a school nurse.

### Demographic factors

Demographic factors were based on the childhood questionnaire data and comprised: immigration background of the child, social class according to the Hall–Jones social scale of occupational prestige (average of parents), log-normalised family income, education level of most educated parent (no qualifications, GCSE or equivalent, A-level of equivalent, higher education) and the number of siblings. Immigration background was coded as a binary variable with 0 if the child’s ethnicity was stated as “English” or “Irish” and as 1 otherwise.

### Analysis

The analyses were carried out using *R* 3.4.3 and *Python* 2.7.11. A full list of the packages used in the analysis is presented in the Supplementary Materials. The code for the analysis is available online (http://www.github.com/joebathelt/). We followed the methods described by [57] so that results can be more easily compared across cohorts.

### Reduction of dimensionality

Exploratory Factor Analysis (EFA) reduced the dimensionality of the questionnaire data. First, we converted the childhood ratings to 3- and 4-point discrete scales using kernel density estimation to make the data comparable between the two time points. Next, we calculated the correlation between questionnaire items separately for each time point. Correlations were based on polychoric correlations in a maximum likelihood procedure [50] using the *polycor* package v0.7.9 under *R* 3.4.3. Next, exploratory factor analysis (EFA) was carried out using the *psych* v1.7.8 package under *R* 3.4.3 with orthogonal factor rotation (*varimax*) to create maximally independent factors. While orthogonal factors may not provide a realistic representation of the latent factors underlying the questionnaire responses, orthogonalisation was necessary for the subsequent clustering analysis that did allow for overlap between the factors. The number of factors was determined through parallel analysis and a bootstrapping procedure (see Supplement for details). Next, univariate (> 3 standard deviations above the mean) and multivariate outliers (Mahalanobis distance > 24.32, see Fig. 1 for attainment) in the factor scores were excluded (see Fig. 1 for attainment).

### Hybrid hierarchical clustering

The identification of subgroups within data is a hard problem with no clear solution [41]. The subgrouping solution will depend on the choice of methodology and the statistical assumptions underlying the method. A plethora of clustering methods has been proposed and a variety of clustering methods have been applied to identify subgroups in mental health research, including latent class analysis, community detection and hierarchical clustering [4, 5, 23, 32, 40, 57]. We employed hierarchical hybrid clustering (HHC) in the current analysis for several reasons. First, we could align our analysis closely with the methods of another large-scale investigation of childhood and adolescent mental health [57]. Keeping the methodology consistent across multiple studies and datasets helps to establish some consistency in the emerging literature on data-driven clustering. Second, developmental accounts suggest that psychopathology differentiates from broader factors of internalising and externalising in early development towards more specific difficulties in later development [2, 36]. A hierarchical clustering approach is well suited to describe hierarchies of overarching dimensions with further subdivisions that may occur at different stages of development. Following the approach of [57], we employed the hierarchical hybrid clustering (HHC) approach developed by Chipman and Tibshirani [9]. This method balances the strength of agglomerative clustering to identify consistent small clusters with the strength of divisive clustering to identify large clusters with broader reach. We used the implementation of the HHC algorithm in the *hybridHclust* v1.0-5 under *R* 3.4.3 (see Supplement for details). To compare our findings to results obtained using an alternative clustering procedure, we present a solution using consensus community detection [38] as previously employed for describing behavioural problems in struggling learners [5] in the Supplementary Materials.

### Assessment of transitions

To assess transitions between childhood and adolescence, we compared the number of individuals transitioning to an equal split using *Χ*^2^ tests, i.e. for 10 clusters, the null model would assume that 10% of children transition to each cluster. Further, the proportion of children being assigned to a cluster in adolescence was compared to an equal split through proportional *z*-tests corrected for multiple comparisons using Bonferroni correction.

### Statistical analysis

Differences between the groups defined by the hierarchical hybrid clustering were investigated in the first instance in two-way analysis of variance (ANOVA) models (group, domain, group x domain). Where Mauchly’s test of sphericity indicated a deviation from the sphericity assumption, Greenhouse–Geisser-corrected values are reported. Follow-up contrasts were based on t-tests relative to the whole sample, i.e. expressing how clustering-defined groups differed from the whole sample. Multiple comparisons arising from the number of factors were adjusted using Bonferroni correction. A significance criterion of *p* < 0.05 was used for all analyses. Effect sizes were expressed as Cohen’s *d*.

We conducted a post hoc power analysis to estimate the minimum sample size to detect a small effect. At significance threshold of *α* < 0.05, a statistical power of 0.95, the minimum sample size required to detect a small effect (Cohen’s *f* ≥ 0.1) with 6 factor domains was *N* = 2093. With the available sample size in the childhood data (*n* = 12,134), an effect size of Cohen’s *f* ≥ 0.04 could be resolved, assuming *α* = 0.05, 1 − *β* = 0.95, and 6 factor domains. With the available data in adolescence (*n* = 6744), an effect size of Cohen’s *f* ≥ 0.06 could be resolved.

## Results

### Dimensionality reduction

**Fig. 2:**
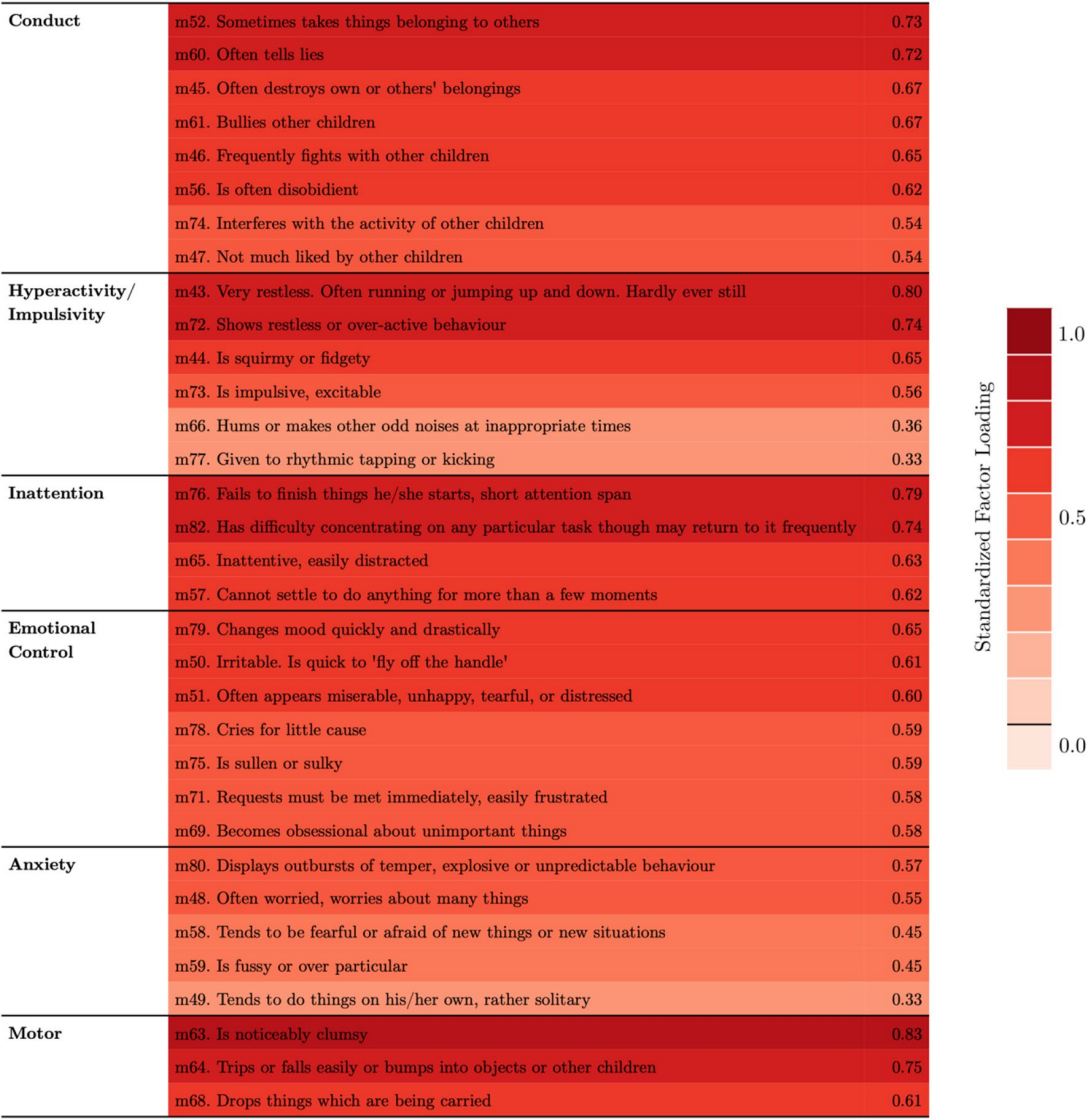
Items associated with each factor in the childhood data. The colour indicates the strength of the standardised factor loading and the numerical values are shown in the second column (see Supplement for the equivalent loading of the items in the adolescence data)

Parallel analysis indicated 9 factors as optimal at both timepoints. The confidence intervals of the bootstrapped factor solution indicated a stable solution with 6 factors (see Fig. 2 for a graphical representation of the factor loading). The 6-factor model explained 56.78% of variance in the childhood data and 53.39% in the adolescence data. We propose the following labels for the 6 factors according to the items associated with them: Conduct problems (C.), Hyperactivity/Impulsivity (H.), Inattention (I.), Emotional control problems (E.), Anxiety (A.), Motor problems (M.). Overviews of the items associated with the factors in the childhood and adolescence data are presented in Fig. 2. The full loading matrix and factor correlation structure can be found in the Supplementary Materials.

### Hybrid hierarchical clustering of the childhood data (10 years)

**Fig. 3:**
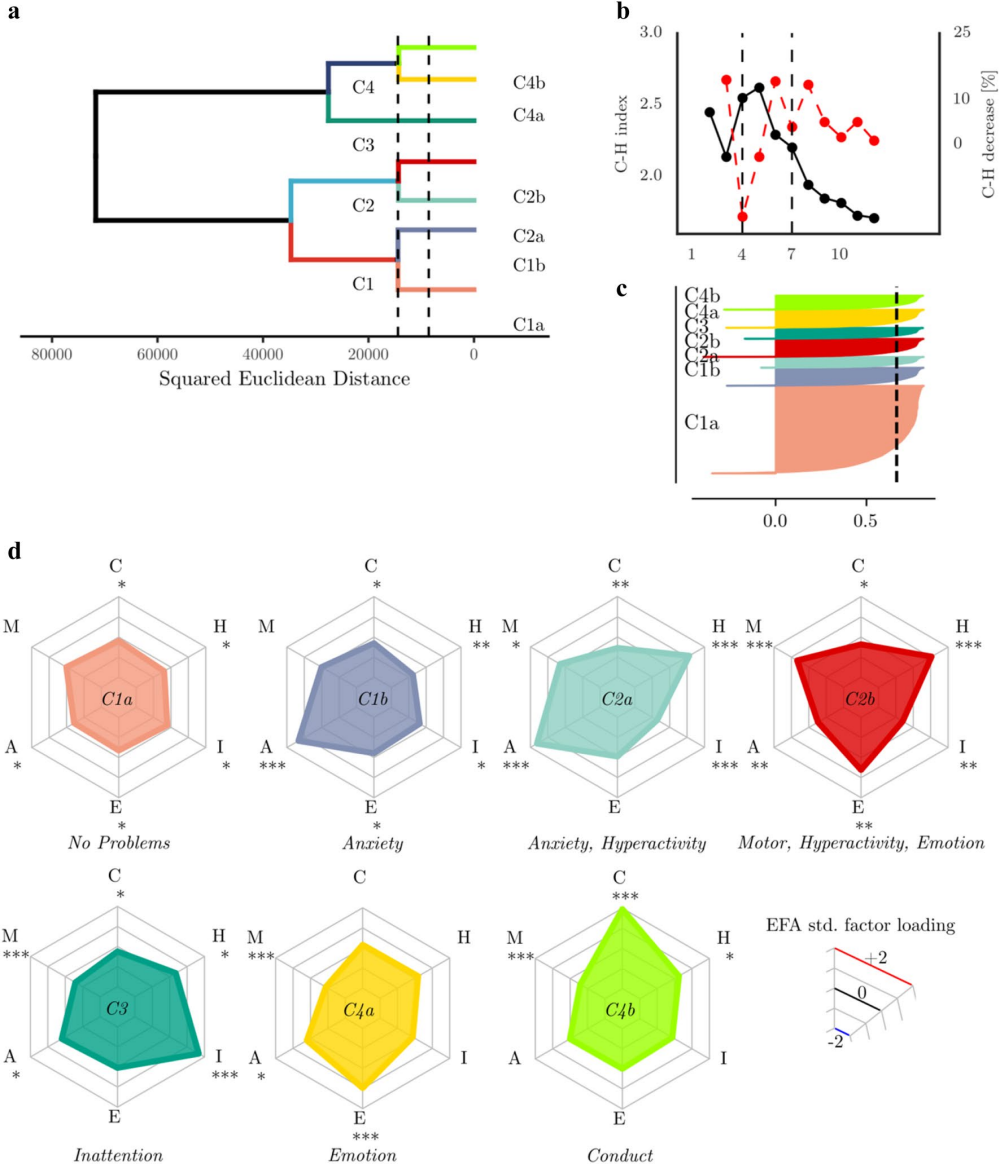
Results of the hybrid hierarchical clustering of the childhood data. **a** Dendrogram of the hierarchical clustering structure. The dashed lines indicate the height cut-off to define 4 and 7 clusters. **b** Calinski–Harabasz index for different cluster numbers. The red line indicates the percentage change from one number of clusters to the next. **c** Silhouette coefficient for each group. The dashed line indicates the average silhouette coefficient across all groups. **d** Profiles of behavioural ratings for each cluster. The asterisks indicate the effect size (Cohen's *d*) relative to the whole sample: *** > 0.8, ** > 0.5, * > 0.2

The hierarchical hybrid clustering algorithm successfully converged on a solution with the childhood data (see Fig. 3). The Calinksi–Harabasz index indicated optimal cluster numbers at *k* = 4 and *k* = 7 (see Fig. 3b). To save space, we focus on the *k* = 7 solution here (a detailed description of the k = 4 solution can be found in the Supplement). The clustering indicated a large group without problems in any domain (C1a ‘*No Problems’*, see Table 2 for descriptive statistics and Fig. 3 for an illustration of the behavioural profiles). Further, groups with problems in specific domains were indicated, which comprised groups with Anxiety problems (C1b ‘*Anxiety*’), Inattention (C3 ‘*Inattention’*), Conduct problems (C4b ‘*Conduct’*), or Emotional problems (C4a ‘*Emotion*’). There were also groups with problems in multiple domains, specifically a group with Anxiety and Hyperactivity (C2a ‘*Anxiety, Hyperactivity’*), and a group with Motor, Hyperactivity and Emotional problems (C2b ‘*Motor, Hyperactivity, Emotion’*).

**Table 2:** Overview of size, gender ratio, cognitive and educational assessment performance and incidence of mental health problems in the clustering-defined groups in the childhood data

		Size		Gender	Cognition		Maths		Reading		Mental health	
		*n*	%	% Female	Mean	SD	Mean	SD	Mean	SD	# Cases	%
C1a	No problems	6006	49.51	48.15	0.07	1.023***	0.2	0.956***	0.2	0.941***	44	0.73
C1b	Anxiety	1228	10.12	49.02	− 0.05	0.953	− 0.22	0.952***	− 0.17	1.006***	97	7.90
C2a	Anxiety, hyperactivity	733	6.11	60.03***	− 0.08	0.928	− 0.37	1.021***	− 0.39	1.038***	36	4.91
C2b	Motor, hyperactity, emotion	1231	6.04	51.75	− 0.02	1.015	− 0.03	0.979	− 0.03	1.007	40	3.25
C3	Inattention	741	10.15	39.27***	− 0.24	0.975***	− 0.43	1.009***	− 0.46	0.967***	44	5.94
C4a	Emotion	1236	7.91	52.59	0.01	0.968	− 0.02	1.018	− 0.03	1.009	55	4.45
C4b	Conduct	959	10.19	44.42**	− 0.12	0.953***	− 0.33	0.97***	− 0.33	0.986***	24	2.50

The percentage of cases with emotional or behavioural problems (mental health) relative to the group size is shown in the last column. Statistical comparison to the whole sample (Bonferroni-corrected): ***p < 0.001, **p < 0.01, *p < 0.05

Regarding differences in other domains, there were more girls than expected by chance in the Inattention cluster (C3 ‘*Inattention’*), and more boys in the Anxiety and Hyperactivity (C2a ‘*Anxiety, Hyperactivity’*) and the Conduct problem cluster (C4b ‘*Conduct*; see Table 2). Children with anxiety and emotional problems (C1b ‘*Anxiety*’, C3 ‘*Inattention*, C4a ‘*Emotion*’) scored lower across multiple measures of family structure and socio-economic background (see Table 2 and Supplement for a detailed analysis). Cognitive scores and educational attainment scores were higher in children with no behavioural problems (C1a ‘*No Problems’*) and lower for children with anxiety, inattention, or conduct problems (C1b ‘*Anxiety’*, C3 ‘*Inattention’*, C4b ‘*Conduct’*, see Table 2 and Supplement). Mental health problems were more frequently indicated for children with anxiety or emotional problems (C1b ‘*Anxiety’*, C4a ‘*Emotion’*).

### Hierarchical hybrid clustering of the adolescence data (16 years)

**Fig. 4:**
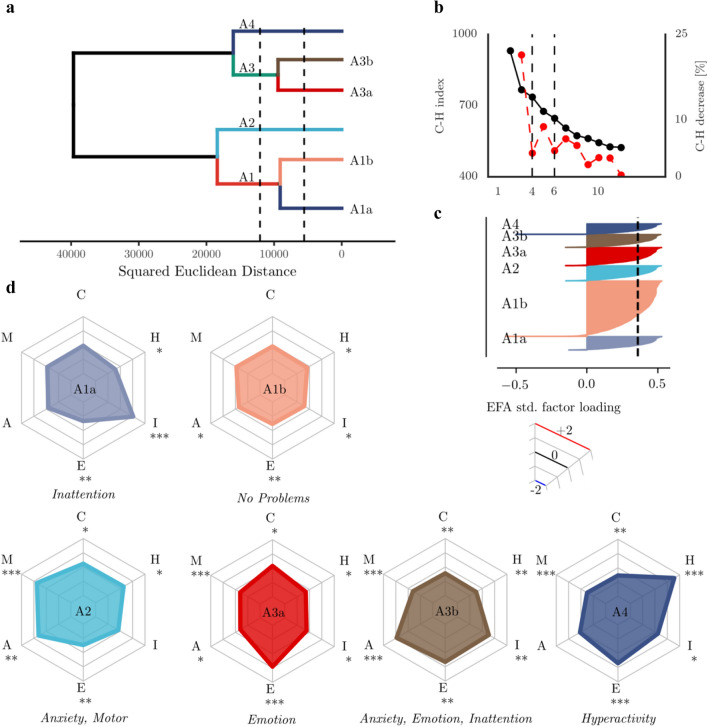
Results of the hybrid hierarchical clustering of the adolescence data. **a** Dendrogram of the hierarchical clustering structure. The dashed lines indicate the height cut-off for 4 and 6 clusters. **b** Calinski–Harabasz index for different cluster numbers. The red line indicates the percentage change from one number of clusters to the next. **c** Silhouette coefficient for each group. The dashed line indicates the average silhouette coefficient across all groups. **d** Profiles of behavioural ratings for each cluster. The asterisks indicate Cohen’s d relative to mu = 0 and the standard deviation across all groups: *** > 0.8, ** > 0.5, * > 0.2

Inspection of the minima of the Calinski–Harabasz index indicated optimal solutions at 4 and 6 clusters (see Fig. 4b). To save space, we focus on the *k* = 6 solution (see Supplement for *k* = 4 results). At *k* = 6, the statistical comparison indicated differences in factor scores between the groups (ANOVA group x factor: *F*(25, 33,690) = 648.96). The clustering solution identified a large group with no particular problems (A1b ‘*No Problems’*, see Table 3 for descriptive statistics and Fig. 4 for an illustration of the behavioural profiles) and a cluster of adolescents with Hyperactivity (A4 ‘*Hyperactivity’*). Further, the clustering pulled out a group with selective Inattention problems (A1a ‘*Inattention*’). In addition, the clustering indicated groups with combinations of problems across domains, i.e. a group with problems with Anxiety and Motor problems (A2 ‘*Anxiety, Motor’*), a group with Emotional problems (A3a ‘*Emotion*’) and a group with problems related to Anxiety, Emotion and Inattention (A3b ‘*Anxiety, Emotion, Inattention’*).

**Table 3:** Overview of size, gender ratio, cognitive and educational assessment performance and incidence of mental health problems in the clustering-defined groups in the adolescence data

		Size		Gender	Cognition		Maths		Reading		Mental health	
		*n*	%	% Female	Mean	SD	Mean	SD	Mean	SD	# Cases	%
A1a	Inattention	721	10.71	55.21***	7.66	3.702	− 0.07	1.048	46.31	23.919	7	0.97
A1b	No problems	2981	44.26	50.72*	7.77	3.677	0.14	0.999***	49.21	23.665	18	0.60
A2	Anxiety, motor	806	11.97	30.02***	7.37	3.862	− 0.06	0.982	45.72	24.529	6	0.74
A3a	Emotion	972	14.43	42.91***	7.54	3.771	0.05	0.895	43.29	24.392	7	0.72
A3b	Anxiety, enotion, inattention	692	10.27	59.68***	7.43	3.739	− 0.41	0.939***	44.06	22.664	8	1.16
A4	Hyperactivity	563	8.36	50.44	7.35	3.749	− 0.19	1.006	43.76	23.488	8	1.42

The percentage of cases with emotional or behavioural problems (mental health) relative to the group size is shown in the last column. Statistical comparison to the whole sample (Bonferroni-corrected): ***p < 0.001, **p < 0.01, *p < 0.05

Regarding differences in other factors, there were more girls in the cluster without behavioural problems (A1b ‘*No Problems*’), in the Inattention (A1a ‘*Inattention*’), and mixed problems cluster (A3b ‘*Anxiety, Emotion, Inattention’*) than would be expected by chance (see Table 3). There were more boys in the Anxiety and Motor problems (A2 ‘*Anxiety, Motor*’) and the Emotional problems cluster (A3a ‘*Emotion*’). There was no difference in cognitive or reading scores between the groups (see Table 3 and Supplement). Scores on the arithmetic assessment were higher for adolescents in the no problems group (A1b ‘*No Problems*’) and lower for adolescents with problems related to Anxiety, Inattention and Emotional Problems (A3b ‘*Anxiety, Emotion, Inattention*’, see Table 3 and Supplement). There were no differences in the frequency of mental health problems between the groups on the school nurse report (although note that only a third of the sample had data available, see Table 3 and Supplement for details and Discussion for limitations associated with this rating).

### Differences in behavioural problem profiles between childhood and adolescence

Of the 6744 adolescents included in the analysis, 5487 participants were also included in the childhood data. Statistical comparison indicated no disproportionate loss per group either defined in the childhood or adolescence data (defined at 6/7 cluster level; childhood grouping: *Χ*^2^(49) = 56, *p* = 0.229; adolescence grouping: *Χ*^2^(25) = 30, *p* = 0.224). The ratings of behavioural difficulties shifted between the assessments at childhood and adolescence. Statistical comparison indicated that the average parent rating of behavioural problems across all items decreased (childhood: mean = 1.31, SE = 0.008; adolescence: mean = 1.27, SE = 0.007; paired-sample *t* test: *t* (5791) = 11.04, *p* < 0.001, *d* = 0.15). The pattern of differences differed by the behavioural domain. The largest change was observed for items related to Hyperactivity with lower ratings for adolescence compared to childhood (10 yo: mean = 1.41, SE = 0.009; 16 yo: mean = 1.24, SE = 0.007; paired-sample t test: t (5791) = 26.61, *p* < 0.001, *d* = 0.35). Smaller decreases were found for Anxiety (childhood: mean = 1.46, SE = 0.011; adolescence: mean = 1.36, SE = 0.007; *t* = 16.37, *p* < 0.001, *d* = 0.22) and Inattention (childhood: mean = 1.43, SE = 0.009; adolescence: mean = 1.36, SE = 0.008; *t* = 10.13, *p* < 0.001, *d* = 0.13). There were smaller decreases for items related to Motor and Conduct Problems (Motor: *d* = 0.05; Conduct: *d* = 0.10). In contrast, significant increases were found for problems related to Emotion (childhood: mean = 1.34, SE = 0.009; adolescence: mean = 1.41, SE = 0.009; *t* = − 11.39, *p* < 0.001; *d* = -0.15). In summary, the overall incidence of behavioural problems and the nature of behavioural problems changed between the childhood and the adolescence assessment.

The changing nature of parent-reported behavioural problems was also reflected in the hybrid hierarchical clustering solution in childhood and adolescence. Comparison of the Fowlkes–Mallows index at *k* = 4 indicated that the groups were not similar (two-sided permutation-*p* = 0.034, mean = 0.36, variance = 4.861 × 10^–10^), and was significantly lower in adolescence compared to a bootstrapped sample of the same index based on random permutations of the childhood dendrogram (two-sided permutation-*p* < 0.001, 1,000 permutations, (see Fig. S3 in the Supplementary Materials). When comparing the dendrograms at the 6-group (childhood) and 7-group cut-off (adolescence), the clustering solution was also dissimilar (mean = 0.25, variance = 5.944 × 10^–6^) and lower than can be expected by chance (two-sided permutation-*p* < 0.001, 1000 permutations, see Supplementary Materials). In short, the profiles of behavioural problems were different in adolescence and childhood.

**Fig. 5:**
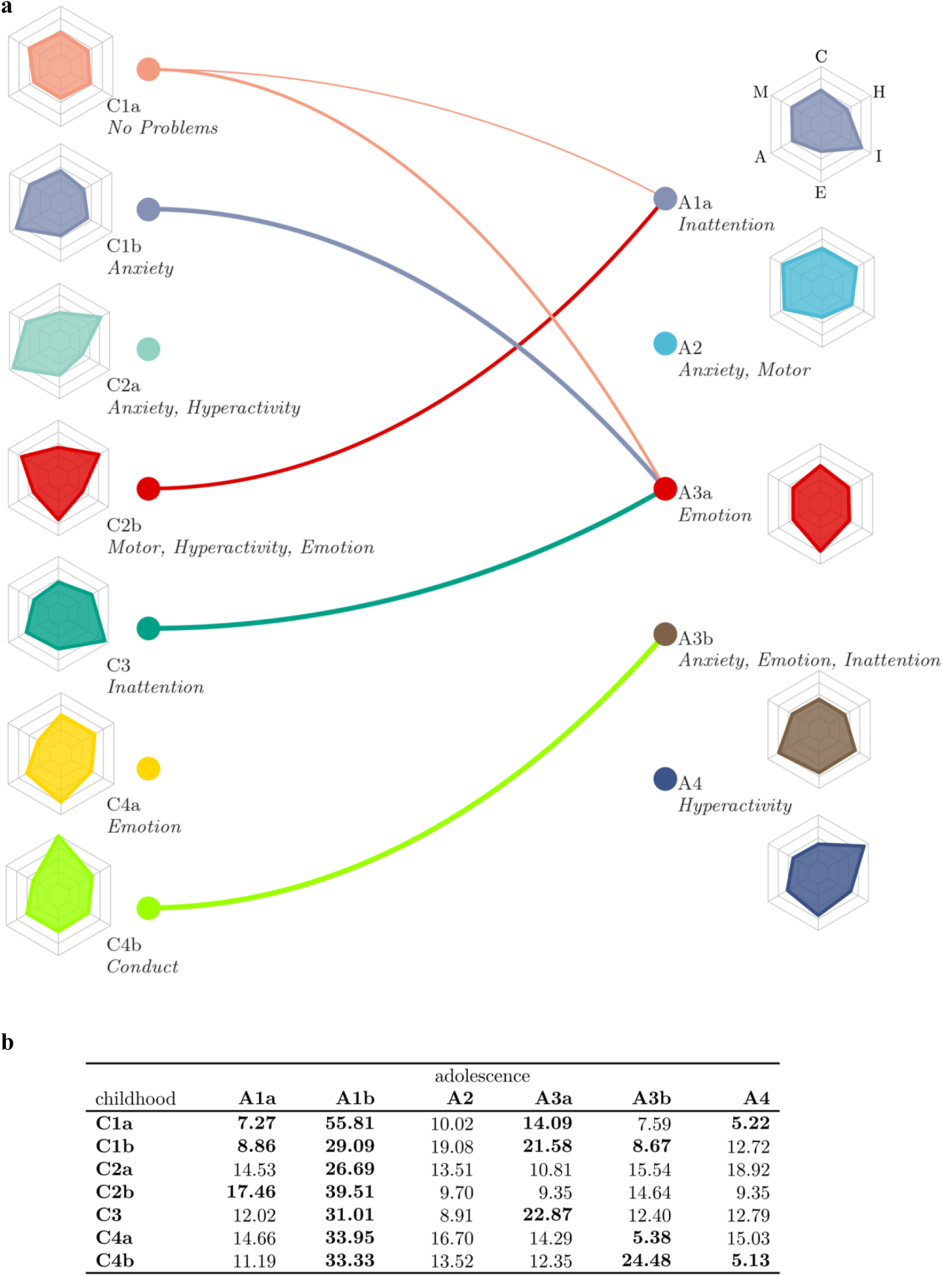
Overview of transitions between childhood and adolescence behavioural problem profiles. **a** Illustration of the transitions. The left side shows groups identified in the childhood data and the right side shows groups in adolescence data. The thickness of the line indicates the proportion of children transferring from the childhood group to the adolescent groups. Only transitions that significantly differed from the proportion expected by chance are shown. Transitions to the typical group (A1b) are omitted from the figure. The mini maps show the behavioural profile of the corresponding groups (see Figs. 3 and 4 for larger versions). **b** Table overview of transitions between childhood and adolescence showing the percentage of children transferring from groups identified in the childhood assessment (rows) to groups in the adolescence assessment (columns). Bold entries indicate proportions that were significantly above or below what would be expected by chance (Bonferroni corrected)

Differences between the clustering at childhood and adolescence were reflected in transitions of individuals between the behavioural difficulty profiles (see Fig. 5). Across all profiles in childhood, a significant proportion transitioned to the low behavioural problems group in adolescence (A1b, see Fig. 5b). There were also transitions to profiles with behavioural problems. Specifically, a larger-than-expected proportion transitioned from no behavioural problems in childhood to Emotion problems (A3a ‘*Emotion*’: see Fig. 5), while a transition to Inattention (A1a ‘*Inattention*’) or Hyperactivity (A4 ‘*Hyperactivity*’) was less common. For children with Anxiety problems (C1b ‘*Anxiety*’), a large proportion showed Emotional problems in adolescence (A3a ‘*Emotional*’). Children with mixed problems related to Emotion, Motor and Hyperactivity (C2b ‘*Motor, Hyperactivity, Emotional’*) mostly transitioned to problems related to Inattention in adolescence (A1a ‘*Inattention*’). Children with Anxiety problems (C3) tended to show problems with Emotion in adolescence (A3a, ‘*Emotion*’). A large number of individuals with Conduct problems in childhood (C4b ‘*Conduct*’) transitioned to mixed problems with Anxiety, Emotion and Inattention (A3b ‘*Anxiety, Emotion, Inattention’*) in adolescence.

Next, we investigated if any of the demographic factors in childhood predicted common transitions between childhood and adolescence. In each case, we compare a group with a particular profile of behavioural problems, e.g. A1a, to the rest of the sample, e.g. A1a^c^. The assessments at childhood were used to assess their impact on problems in adolescence. Children who transitioned to the no behavioural problems cluster in adolescence (A1b, ‘*No Problems’*) from childhood clusters with some indication of problems, i.e. any of the childhood clusters except C1a (‘*No Problems*’), came from families with higher income (mean_A1b_ = 1.38, SE_A1b_ = 0.007; mean_A1b_^c^ = 1.34, SE_A1b_^c^ = 0.006, *t* (5060) = 4.34, *p* < 0.001), more educated parents (mean_A1b_ = 2.17, SE_A1b_ = 0.021; mean_A1b_^c^ = 2.08, SE_A1b_^c^ = 0.018, *t* (5371) = 2.91, *p* = 0.018) and higher social class (mean_A1b_ = 3,36, SE_A1b_ = 0.024; mean_A1b_^c^ = 3.55, SE_A1b_^c^ = 0.019, *t* (5325) = −  6.21, *p* < 0.001). Children who overcame behavioural problems by age 16 also had better cognitive scores in childhood (mean_A1b_ = 0.21, SE_A1b_ = 0.020; mean_A1b_^c^ = 0.09, SE_A1b_^c^ = 0.018, *t* (5480) = 4.76, *p* < 0.001). Immigration background and the number of siblings were not related to the transition to low-behavioural problems (all *p* > 0.1).

Transition to the cluster with problems with Emotions (A3a ‘*Emotion*’) from any of the childhood clusters was predicted by lower family income (mean_A3a_ = 1.32, SE_A3a_ = 0.012; mean_A3a_^c^ = 1.37, SE_A3a_^c^ = 0.005, *t* (5066) = − 3.52, *p* = 0.002), a greater number of siblings (mean_A3a_ = 2.65, SE_A3a_ = 0.0,039; mean_A3a_^c^ = 2.49, SE_A3a_^c^ = 0.015, *t* (5452) = − 2.26, *p* < 0.001) and lower social class (mean_A3a_ = 3.64, SE_A3a_ = 0.041; mean_A3a_^c^ = 3.43, SE_A3a_^c^ = 0.017, *t* (5066) = − 3.52, *p* = 0.002). Immigration background, highest parental education and childhood cognitive scores were not related to the transition to Emotion problems in adolescence (all *p* > 0.1).

## Discussion

Understanding the developmental context of behavioural problems is a crucial missing piece in current diagnostic systems like DSM and ICD [48]. Despite the well-documented changes to cognitive and social development between childhood and adolescence, the most common profiles of behavioural problems at both stages remained unclear. Using data-driven clustering in population-representative birth cohort, we identified groups with relatively selective problems related to anxiety, inattention and conduct/emotion. Other groups contained a constellation of problems related to hyperactivity, inattention and motor problems. By comparing group membership across time points, we established the most common transitions between childhood and adolescence. The most common transition was towards no behavioural problems. In addition, specific transitions in behavioural problems between childhood and adolescence were observed. These were transitions from inattention and anxiety problems towards problems with emotion, from conduct towards a constellation of problems with anxiety, inattention and emotion, and from motor problems, hyperactivity and emotion towards inattention. In the following sections, we discuss the most common behavioural problems identified in childhood and characterise their transition in adolescence.

### Anxiety/worrying

Problems with anxiety and worrying are among the most prevalent concerns in childhood [13, 22, 39, 54]. The current analysis shows that these problems can either occur in relative isolation or in combination with problems related to inattention or hyperactivity, consistent with studies in diagnostic groups [29, 43].

While a majority of children who were rated as having problems related to anxiety in childhood did not display any behavioural problems in adolescence, a subset showed consistent transitions from anxiety to problems with emotion in adolescence. Previous studies using diagnostic criteria indicated that the prevalence of anxiety disorders follows a U-shaped development with higher prevalence in late childhood, then a decline during mid-adolescence (15–16 years), and a subsequent rise in late adolescence (> 17 years) and early adulthood [13, 22, 58]. The current study provides an alternative explanation by suggesting that for some children, problems with anxiety in childhood may manifest as emotional problems in adolescence. In short, childhood anxiety problems may either dissipate or manifest as emotional dysregulation in adolescence.

### Conduct

Problems with conduct, characterised by delinquency and aggression, have been recognised as a separate group of behavioural problems since DSM-III [46]. While the proportion of 10-year-olds that meet DSM criteria for conduct disorder is only around 3% [62], epidemiological studies found that milder problems with conduct occur in around 35% of children, consistent with the prevalence observed in the current data-driven analysis [49]. While conduct problems emerged as a specific concern in a subgroup of children, conduct problems did not occur as a distinct subgroup in adolescence. A significant proportion of children with conduct problems transitioned to complex problems with anxiety, inattention and emotion in adolescence indicating that childhood conduct problems may be a precursor or manifestation of broader problems. A link between childhood conduct problems and psychopathology has also been established in longitudinal analyses showing that childhood conduct problems are associated with depression, anxiety and social isolation at 18 years Moffitt et al. [45] and, conversely, that 18 year-olds with depression frequently had a history of childhood conduct problems (43%, [56]). In short, conduct problems can either be limited to childhood (33.33%) or can be precursors of problems related to anxiety and emotional dysregulation in adolescence (24.48%).

### Hyperactivity/impulsivity

Problems with the control of attention, regulation of activity levels and the ability to control impulses are recognised as attention-deficit hyperactivity disorder (ADHD) in the DSM or hyperkinetic disorder in ICD-10. Studies of ADHD suggest a high degree of comorbidity, particularly with learning problems, conduct and anxiety [51]. Overlap with conduct problems and anxiety was also indicated in the current analysis but only during childhood. This suggests that the overlap of hyperactivity/impulsivity with other difficulties may be specific to childhood. Children with hyperactivity/impulsivity mostly transitioned to problems with anxiety and emotion in adolescence.

### Motor problems

Problems with motor control are represented in the diagnosis of developmental coordination disorder (DCD) that has been included in the DSM since its third edition. However, there has been a longstanding debate about the selectivity of motor deficits due to the considerable overlap with behavioural difficulties [8, 65]. The current analysis identified overlap with hyperactivity and with anxiety and inattention consistent with studies focused on diagnostic criteria [28, 31]. A large proportion of children with a profile of problems related to motor problems and concomitant hyperactivity and emotion problems transitioned to problems with inattention in adolescence. This may indicate a shift towards milder or more cognitive problems.

### Transition to low behavioural problems

Despite the notion of adolescence as a time of “storm and stress”, the overall ratings of behavioural problems were lower in adolescence compared to childhood. Further, the majority of children with behavioural problems in childhood did not show problems in adolescence (55%). There may be several factors contributing to the reduction in behavioural problems in adolescence. Environmental factors may have helped children to overcome challenges. Consistent with this interpretation, indicators of better family resources (higher family income, education and social class) were related to fewer behavioural problems in adolescence (also see [52] for a review).^2^ In addition, children may have been able to compensate for vulnerabilities over time. The transition to low behavioural problems was associated with better cognitive performance in childhood in line with previous studies that linked cognitive ability with better mental health outcomes across the lifespan [60, 63].

### Emotional dysregulation

Emotional dysregulation has been characterised as a transdiagnostic risk factor in adolescence that has been linked to aggression, anxiety and eating pathology Herts et al. [30]; McLaughlin et al. [44]. The current analysis further suggests that multiple profiles of behavioural problems in childhood converge on emotional dysregulation during adolescence. This may reflect a true convergence by which various vulnerabilities culminate in a common set of symptoms. Alternatively, the repertoire to express behavioural problems may shrink due to different societal expectations and growing independence from parents. Therefore, the outbursts associated with emotional dysregulation may be outward manifestations that are driven by various underlying problems, such as impulsivity [24] or aggression Sourander and Helstelä [55], that are harder to differentiate in adolescence.

### Limitations and intergenerational future directions

There are some limitations to the generalisability of the findings from the current analysis that are important to keep in mind. The items on the behavioural problem ratings may not have captured certain behavioural problems. For instance, items relating to depression were more limited than we would have liked. Consequently, behavioural problems associated with depression may not have been adequately represented in the clustering analysis. In addition, even though the current analysis was based on a cohort study that was designed to be representative of the population, differential attrition between sweeps of data collection may have introduced a bias, i.e. children with fewer behavioural problems were more likely to provide data in adolescence (for a comprehensive discussion of this issue in the BCS data, see [34] and [47]. Consequently, the current analysis may underestimate the prevalence of behavioural difficulties in adolescence. In addition, no direct assessments of mental health from specialist professionals like psychiatrists, psychologists, or mental health nurses were available. Instead, the reports of mental health problems were obtained from school nurses. These reports seem to underestimate the incidence of mental health problems and had high levels of missing data. Adolescents filled in a health questionnaire that contained a question asking how often they felt anxious, depressed, or unhappy in the last 12 months. However, only a fraction of adolescents provided responses for this item (~ 24%). For a comprehensive analysis, dedicated questionnaires about mental health or structured interviews will be needed. Further, the current analysis is based on parent ratings. Teacher ratings were obtained as part of the BCS70 study but were only available for a small subset of adolescents because of a teacher strike in 1986. Parent and teacher ratings provide a different perspective of a young person’s behaviour (e.g. see [20]) and may produce different behavioural profiles. Future  analyses based on studies that obtained parent and teacher ratings will need to investigate this possibility.

There are also some limitations to the analysis approach taken in the study. First, we employed factor rotation to create orthogonal factors as part of the dimensionality reduction step. This is important within our analysis, because when behavioural symptoms co-occur within a cluster, this can be meaningfully interpreted, rather than reflecting the correlations between those dimensions within the overall sample. However, orthogonal factors may not provide a realistic account of the factor structure of the questionnaire itself. Second, the hybrid hierarchical clustering created hard cluster assignments, i.e. each participant was assigned to only one cluster. Soft clustering algorithms or latent variable models that allow participants to be allocated to multiple overlapping clusters may provide a promising avenue for future research.

A crucial next question is whether and how these profiles and transitions have changed across generations. This will provide valuable insight into potential mechanisms underlying much more recent changes in the prevalence of adolescent mental ill-health. A study comparing the prevalence of parent-reported behavioural problems between the BCS cohort from 1970 and a cohort from 2006 indicated higher rates of behavioural problems in the 2006 cohort [11]. This may reflect an increasing awareness of behavioural problems in society or a greater prevalence of behavioural problems due to changing environmental factors that influence behavioural problems, e.g. influence of cyber-bullying [1]. Future epidemiological studies that incorporate more comprehensive mental health assessments are now needed to corroborate the findings of the current analysis and establish whether and how these transitions have changed intergenerationally. The data-driven mapping process that we demonstrate provides an ideal way of sidestepping ongoing changes in diagnostic criteria.

## Conclusion

We used data-driven hierarchical clustering to identify common behavioural profiles in childhood and adolescence, map transitions across a 6-year period, and identify the factors that predict specific transitions into adolescence at a population level. Over half of all children that experienced behavioural problems in childhood did not exhibit any behavioural problems as adolescents. These children can be predicted by their cognitive ability and family resources. There were also some key transitions. Multiple problems that are relatively common such as anxiety, conduct problems, and hyperactivity converged on problems with emotion in adolescence, while motor problems shifted towards inattention in adolescence. The transition of profiles between childhood and adolescence—hitherto undocumented—highlights that profiles of behavioural problems may be substantially different at developmental phases. Current efforts to move to a proactive model of mental health and wellbeing will likely depend upon recognising these shifts in the presentation of behavioural problems across development.

## Supplementary Information

Below is the link to the electronic supplementary material.

Supplementary file1 (DOCX 1468 KB)

## Footnotes

1 However, the proportions of students attending schools within each sector are broadly representative, albeit with a slight under-representation of Private and Secondary Modern students, as shown in Table 10. See data note below and section on reliability. https://cls.ucl.ac.uk/wp-content/uploads/2017/07/BCS70-Data-Note-1-16-year-Head-Teacher-Questionnaire.pdf

2 Please note that causality could also flow in the opposite direction, i.e. children with more behavioural problems could be a strain on the parents’ financial resources.
