# Supplementary material for: Just a phase? Mapping the transition of behavioural problems from childhood to adolescence
Source: Soc Psychiatry Psychiatr Epidemiol. 2021 Feb 11;56(5):821–36. doi: 10.1007/s00127-020-02014-4 (PMC8068698; doi:10.1007/s00127-020-02014-4)
Supplement: Supplementary file 1 — Supplementary file1 (DOCX 1468 KB) [file 127_2020_2014_MOESM1_ESM.docx]

# Questionnaire items

**Table S1** Items and item codes for the childhood data

| Item code | Item content |
| --- | --- |
| m43. | Very restless. Often running or jumping up and down. Hardly ever still |
| m44. | Is squirmy or fidgety |
| m45. | Often destroys own or others' belongings |
| m46. | Frequently fights with other children |
| m47. | Not much liked by other children |
| m48. | Often worried, worries about many things |
| m49. | Tends to do things on his/her own, rather solitary |
| m50. | Irritable. Is quick to 'fly off the handle' |
| m51. | Often appears miserable, unhappy, tearful, or distressed |
| m52. | Sometimes takes things belonging to others |
| m53. | Has twitches, mannerisms, or tics of the face or body |
| m54. | Frequently sucks thumb or finger |
| m55. | Frequently bites nails or fingers |
| m56. | Is often disobedient |
| m57. | Cannot settle to do anything for more than a few moments |
| m58. | Tends to be fearful or afraid of new things or new situations |
| m59. | Is fussy or over particular |
| m60. | Often tells lies |
| m61. | Bullies other children |
| m63. | Is noticeably clumsy |
| m64. | Trips or falls easily or bumps into objects or other children |
| m65. | Inattentive, easily distracted |
| m66. | Hums or makes other odd noises at inappropriate times |
| m67. | Has difficulty picking up small objects |
| m68. | Drops things which are being carried |
| m69. | Becomes obsessional about unimportant things |
| m71. | Requests must be met immediately, easily frustrated |
| m72. | Shows restless or over-active behaviour |
| m73. | Is impulsive, excitable |
| m74. | Interferes with the activity of other children |
| m75. | Is sullen or sulky |
| m76. | Fails to finish things he/she starts, short attention span |
| m77. | Given to rhythmic tapping or kicking |
| m78. | Cries for little cause |
| m79. | Changes mood quickly and drastically |
| m80. | Displays outbursts of temper, explosive or unpredictable behaviour |
| m81. | Has difficulty using scissors |
| m82. | Has difficulty concentrating on any particular task though may return to it frequently |

**Table S2** Items and item codes for the adolescence data

| Item code | Item content |
| --- | --- |
| pa5.1 | Very restless, Often running about or jumping up and down, Hardly ever still |
| pa5.2 | Is squirmy/fidgety |
| pa5.3 | Often destroys things belonging to others |
| pa5.4 | Frequently fights with others |
| pa5.5 | Not much liked by others |
| pa5.6 | Often worried worries about many things |
| pa5.7 | Tends to do things on own rather solitary |
| pa5.8 | Irritable, Is quick to fly off the handle |
| pa5.9 | Often appears miserable unhappy tearful or distressed |
| pa5.10 | Sometimes takes things belonging to others |
| pa5.11 | Has twitches mannerisms or tics of the face or body |
| pa5.12 | Frequently sucks thumbs or fingers |
| pa5.13 | Frequently bites nails or fingers |
| pa5.14 | Is often disobedient |
| pa5.15 | Cannot settle to anything for more than a few moments |
| pa5.16 | Tends to be fearful or afraid of new things or new situations |
| pa5.17 | Is fussy or overparticular |
| pa5.18 | Often tells lies |
| pa5.19 | Bullies others |
| pa6.1 | Is noticeably clumsy |
| pa6.2 | Trips or falls easily or bumps into objects and other people |
| pa6.3 | Inattentive easily distracted |
| pa6.4 | Hums or makes other odd noises at inappropriate times |
| pa6.5 | Has difficulty picking up small objects |
| pa6.6 | Drops things which are being carried |
| pa6.7 | Becomes obsessional about unimportant things |
| pa6.8 | Requests must be met immediately |
| pa6.9 | Shows restless or over-active behaviour |
| pa6.10 | Is impulsive excitable |
| pa6.11 | Interfered with the activity of others |
| pa6.12 | Is sullen or sulky |
| pa6.13 | Fails to finish things he/she started, Short attention span |
| pa6.14 | Given to rhythmic tapping or kicking |
| pa6.15 | Cries for little cause |
| pa6.16 | Changes mood quickly and drastically |
| pa6.17 | Displays outbursts of temper, explosive or unpredictable behaviour |
| pa6.18 | Has difficulty in using scissors |
| pa6.19 | Has difficulty concentrating on any particular task though may return to it frequently |

# Item-by-item correlation matrices


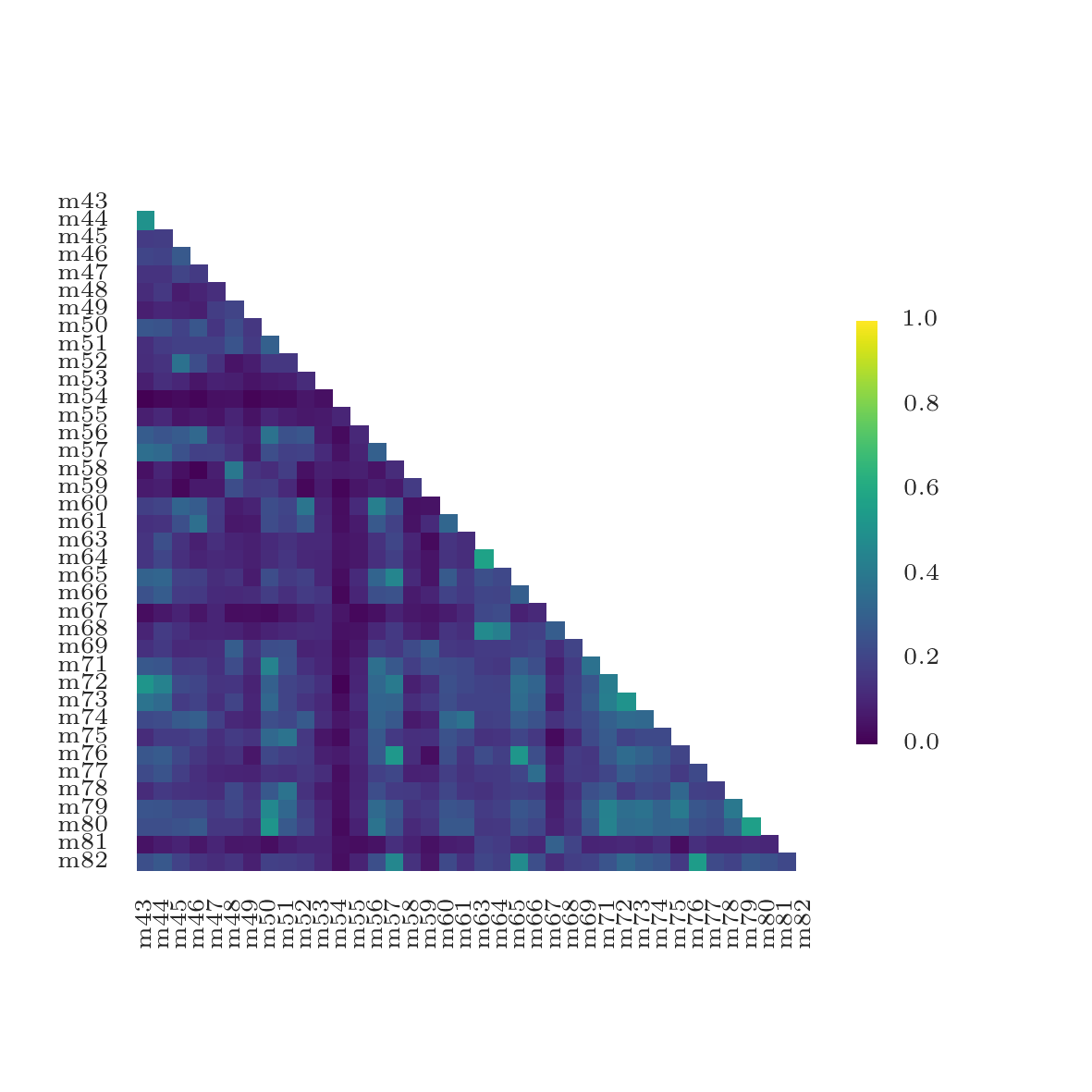


**Figure S1** Item-by-item correlation matrix for the childhood data. The colour scale indicates the correlation coefficient for polychoric correlations.


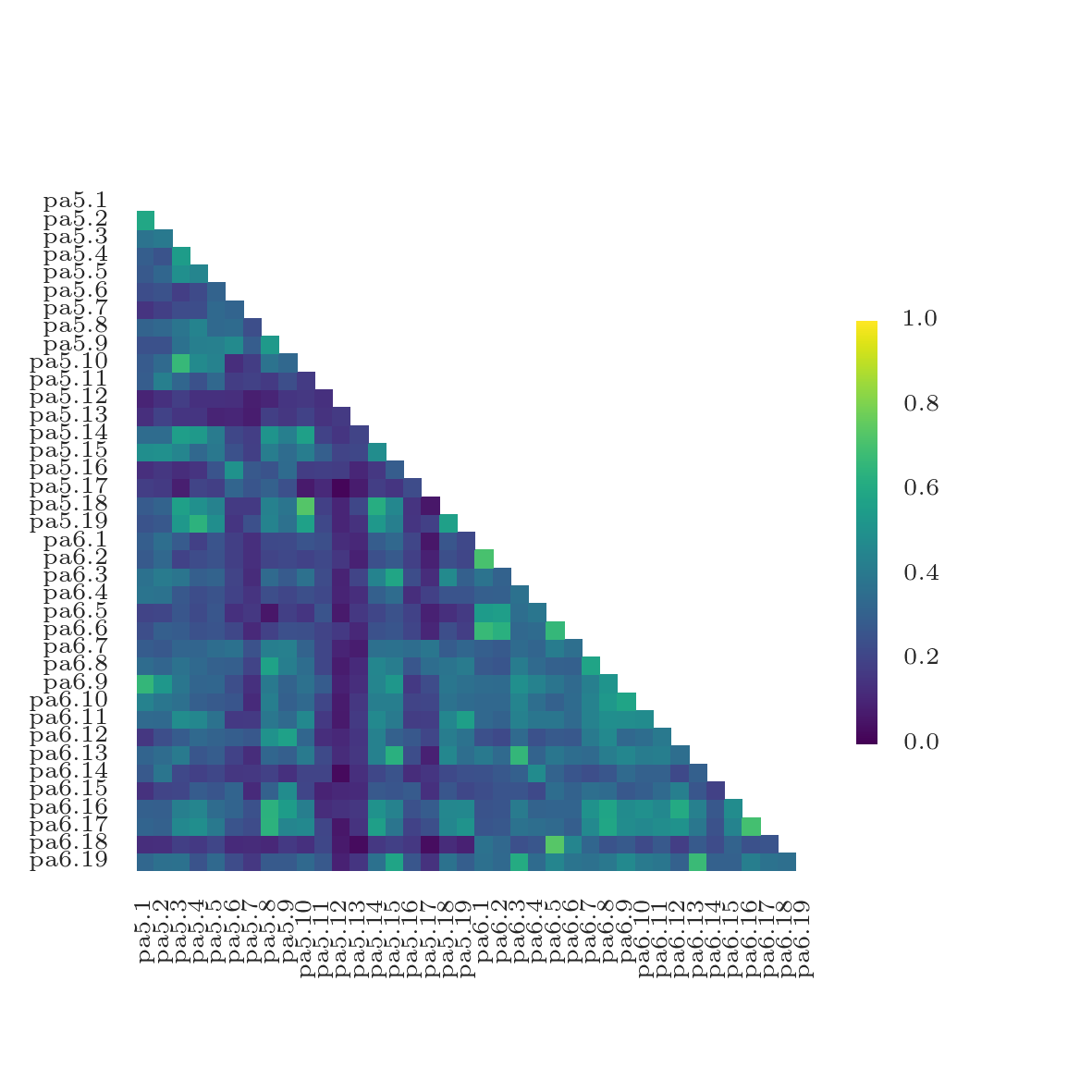


**Figure S2** Item-by-item correlation matrix for the adolescence data. The colour scale indicates the correlation coefficient for polychoric correlations.

# Educational assessments

**Childhood data:** The reading assessments consisted of the Shortened Edinburgh Reading Test (ERT) covering vocabulary, syntax, sequencing, comprehension, and retention

(HodderStoughton, 1979). The maths assessment consisted of the Friendly Maths test that assessed knowledge of basic arithmetic operations, understanding of numbers (fractions, percentages, approximation etc.), understanding of measurement (time, length, area, temperature, money etc.), algebra (operations, logic), geometry (shape, angles, symmetry etc.), and statistics (tables, graphs).

**Adolescence data:** The reading assessment consisted of the Shortened Edinburgh Reading Test (ERT) with tasks comprising skimming, vocabulary, reading for facts, points of view, and comprehension. Arithmetic was assessed on the multiple-choice version of the Applied Psychology Unit (APU) Arithmetic Test version of 1986 (Closs & Hutchings, 1976).

# Exploratory Factor Analysis

In childhood three items did not correlate with any item at 0.3 or above (m53. “Has twitches, mannerisms, or tics of the face or body”, m54. “Frequently sucks thumb or finger”, m55. “Frequently bites nails or fingers”, see Supplementary Materials - Item-by-item correlation matrices). The corresponding items were also only weakly correlated (<0.3) in the adolescence data (pa5.11 “Has twitches mannerisms or tics of the face or body”, pa5.12 “Frequently sucks thumbs or fingers”, pa5.13 “Frequently bites nails or fingers”). These items were therefore excluded from the factor analysis for both timepoints.

The number of factors was estimated via parallel analysis that compared the scree plot of the observed data to a bootstrapped sample of 10,0000 permutations

(Zwick, 1986). Further, confidence intervals of factor scores were calculated through bootstrap resampling (10,000 repetitions) to estimate the reliability of factor loadings. Confidence intervals overlapping with zero indicate an unstable clustering solution. Therefore, the number of factors was reduced if the confidence interval of factor loadings overlapped with zero for a large proportion (>0.5) of items on any of the factors (Van Dam et al., 2016).

# Factor loadings and factor-by-factor correlation matrices

**Figure 3** Items associated with each factor in the adolescence data. The colour indicates the strength of the standardised factor loading.

**Table S3** Standardised factor loadings for the childhood data. The last two rows list the sum of eigenvalues and the proportion of variance explained by each factor respectively. Factor labels: E. Emotional Control, C. Conduct, I. Inattention, H. Hyperactivity/Impulsivity, M. Motor control A. Anxiety

|  | C. | E. | H. | M. | A. | I. |
| --- | --- | --- | --- | --- | --- | --- |
| m43 | 0.22 | 0.09 | 0.80 | -0.04 | 0.19 | 0.08 |
| m44 | 0.22 | 0.16 | 0.65 | -0.01 | 0.24 | 0.24 |
| m45 | 0.67 | 0.13 | 0.18 | 0.16 | 0.21 | 0.11 |
| m46 | 0.65 | 0.18 | 0.22 | -0.02 | 0.06 | 0.05 |
| m47 | 0.38 | 0.21 | 0.11 | 0.24 | 0.10 | 0.13 |
| m48 | -0.02 | 0.55 | 0.10 | 0.03 | 0.13 | 0.07 |
| m49 | 0.12 | 0.33 | 0.04 | 0.01 | 0.01 | 0.10 |
| m50 | 0.40 | 0.61 | 0.30 | -0.07 | 0.06 | 0.01 |
| m51 | 0.34 | 0.60 | 0.06 | 0.00 | 0.15 | 0.14 |
| m52 | 0.73 | 0.06 | 0.09 | 0.19 | 0.19 | 0.08 |
| m56 | 0.22 | 0.11 | 0.14 | 0.40 | 0.09 | 0.11 |
| m57 | 0.62 | 0.30 | 0.31 | -0.08 | 0.19 | 0.06 |
| m58 | 0.31 | 0.14 | 0.38 | 0.17 | 0.62 | 0.10 |
| m59 | -0.07 | 0.45 | -0.02 | 0.17 | 0.18 | 0.07 |
| m60 | 0.00 | 0.45 | 0.12 | 0.23 | -0.08 | -0.08 |
| m61 | 0.72 | 0.15 | 0.12 | 0.12 | 0.19 | 0.10 |
| m63 | 0.67 | 0.24 | 0.15 | 0.28 | 0.05 | 0.06 |
| m64 | 0.13 | 0.16 | 0.16 | 0.25 | 0.19 | 0.83 |
| m65 | 0.15 | 0.18 | 0.17 | 0.32 | 0.12 | 0.75 |
| m66 | 0.28 | 0.16 | 0.30 | 0.14 | 0.63 | 0.17 |
| m67 | 0.26 | 0.17 | 0.36 | 0.29 | 0.19 | 0.17 |
| m68 | 0.17 | 0.10 | 0.04 | 0.91 | 0.09 | 0.35 |
| m69 | 0.16 | 0.19 | 0.15 | 0.49 | 0.13 | 0.61 |
| m71 | 0.12 | 0.58 | 0.20 | 0.36 | 0.07 | 0.11 |
| m72 | 0.24 | 0.58 | 0.38 | 0.21 | 0.14 | 0.02 |
| m73 | 0.26 | 0.24 | 0.74 | 0.28 | 0.24 | 0.05 |
| m74 | 0.23 | 0.37 | 0.56 | 0.15 | 0.20 | 0.09 |
| m75 | 0.54 | 0.23 | 0.27 | 0.32 | 0.18 | 0.11 |
| m76 | 0.38 | 0.59 | 0.06 | -0.12 | 0.12 | 0.15 |
| m77 | 0.23 | 0.18 | 0.23 | 0.07 | 0.79 | 0.14 |
| m78 | 0.25 | 0.20 | 0.33 | 0.24 | 0.20 | 0.14 |
| m79 | 0.26 | 0.59 | 0.03 | 0.11 | 0.15 | 0.12 |
| m80 | 0.37 | 0.65 | 0.27 | 0.13 | 0.15 | 0.06 |
| m81 | 0.44 | 0.57 | 0.27 | 0.20 | 0.09 | 0.02 |
| m82 | 0.12 | 0.14 | -0.01 | 0.62 | 0.27 | 0.21 |
| SS load. | 4.86 | 4.53 | 3.37 | 2.81 | 2.73 | 2.13 |
| Prop. exp. | 0.14 | 0.13 | 0.09 | 0.08 | 0.08 | 0.06 |

**Table S4** Factor-by-factor correlation matrix for the childhood data after varimax factor rotation. Factor labels: E. Emotional Control, C. Conduct, I. Inattention, H. Hyperactivity/Impulsivity, G. Gross motor control A. Anxiety, F. Fine motor control

|  | C. | E. | H. | M. | A. | I. |
| --- | --- | --- | --- | --- | --- | --- |
| C. | 1.00 | 0.07 | 0.05 | 0.02 | 0.04 | 0.00 |
| E. | 0.07 | 1.00 | 0.05 | 0.00 | 0.01 | 0.01 |
| H. | 0.05 | 0.05 | 1.00 | -0.02 | 0.06 | 0.01 |
| M. | 0.02 | 0.00 | -0.02 | 1.00 | 0.00 | 0.06 |
| A. | 0.04 | 0.01 | 0.06 | 0.00 | 1.00 | 0.03 |
| I. | 0.00 | 0.01 | 0.01 | 0.06 | 0.03 | 1.00 |

**Table S5** Standardised factor loadings for the adolescence data. The last two rows list the sum of eigenvalues and the proportion of variance explained by each factor respectively. Factor labels: E. Emotional Control, C. Conduct, I. Inattention, H. Hyperactivity/Impulsivity, M. Motor control A. Anxiety

|  | **C.** | **E.** | **M.** | **H.** | **I.** | **A.** |
| --- | --- | --- | --- | --- | --- | --- |
| pa5.1 | 0.17 | 0.14 | 0.09 | 0.77 | 0.12 | 0.09 |
| pa5.2 | 0.23 | 0.06 | 0.11 | 0.65 | 0.18 | 0.20 |
| pa5.3 | 0.70 | 0.12 | 0.17 | 0.24 | 0.14 | 0.10 |
| pa5.4 | 0.63 | 0.27 | 0.14 | 0.13 | -0.03 | 0.17 |
| pa5.5 | 0.51 | 0.10 | 0.20 | 0.17 | 0.07 | 0.36 |
| pa5.6 | 0.03 | 0.22 | 0.06 | 0.15 | 0.07 | 0.68 |
| pa5.7 | 0.19 | 0.12 | 0.12 | 0.07 | -0.03 | 0.40 |
| pa5.8 | 0.34 | 0.63 | -0.09 | 0.20 | 0.12 | 0.27 |
| pa5.9 | 0.31 | 0.44 | 0.07 | 0.05 | 0.09 | 0.51 |
| pa5.10 | 0.77 | 0.14 | 0.06 | 0.13 | 0.21 | 0.04 |
| pa5.14 | 0.59 | 0.35 | 0.08 | 0.21 | 0.22 | 0.08 |
| pa5.15 | 0.31 | 0.18 | 0.11 | 0.39 | 0.54 | 0.19 |
| pa5.16 | 0.03 | 0.14 | 0.13 | 0.03 | 0.18 | 0.58 |
| pa5.17 | -0.01 | 0.33 | 0.02 | 0.18 | -0.05 | 0.31 |
| pa5.18 | 0.72 | 0.21 | 0.03 | 0.12 | 0.31 | 0.06 |
| pa5.19 | 0.67 | 0.29 | 0.07 | 0.13 | 0.09 | 0.09 |
| pa6.1 | 0.17 | 0.04 | 0.51 | 0.25 | 0.22 | 0.17 |
| pa6.2 | 0.12 | 0.07 | 0.53 | 0.24 | 0.14 | 0.16 |
| pa6.3 | 0.24 | 0.21 | 0.22 | 0.28 | 0.62 | 0.07 |
| pa6.4 | 0.14 | 0.21 | 0.31 | 0.39 | 0.14 | 0.06 |
| pa6.5 | 0.05 | 0.15 | 0.96 | 0.09 | 0.11 | 0.04 |
| pa6.6 | 0.14 | 0.11 | 0.64 | 0.17 | 0.13 | 0.16 |
| pa6.7 | 0.16 | 0.47 | 0.31 | 0.19 | 0.11 | 0.30 |
| pa6.8 | 0.21 | 0.61 | 0.16 | 0.26 | 0.19 | 0.15 |
| pa6.9 | 0.20 | 0.36 | 0.25 | 0.64 | 0.23 | 0.01 |
| pa6.10 | 0.20 | 0.43 | 0.18 | 0.42 | 0.23 | 0.06 |
| pa6.11 | 0.44 | 0.36 | 0.29 | 0.24 | 0.16 | 0.02 |
| pa6.12 | 0.27 | 0.55 | 0.16 | -0.02 | 0.17 | 0.27 |
| pa6.13 | 0.25 | 0.20 | 0.26 | 0.17 | 0.73 | 0.07 |
| pa6.14 | 0.12 | 0.15 | 0.25 | 0.31 | 0.15 | 0.07 |
| pa6.15 | 0.13 | 0.42 | 0.28 | -0.01 | 0.11 | 0.28 |
| pa6.16 | 0.32 | 0.71 | 0.16 | 0.11 | 0.19 | 0.18 |
| pa6.17 | 0.41 | 0.65 | 0.20 | 0.16 | 0.10 | 0.09 |
| pa6.18 | 0.04 | 0.15 | 0.72 | 0.02 | 0.08 | 0.04 |
| pa6.19 | 0.18 | 0.19 | 0.34 | 0.22 | 0.61 | 0.13 |
| SS load. | 4.45 | 3.88 | 3.48 | 2.91 | 2.34 | 2.14 |
| Prop. exp. | 0.12 | 0.11 | 0.09 | 0.08 | 0.07 | 0.06 |

**Table S6** Factor-by-factor correlation matrix for the adolescence data after varimax factor rotation. Factor labels: E. Emotional Control, C. Conduct, I. Inattention, H. Hyperactivity/Impulsivity, G. Gross motor control A. Anxiety, F. Fine motor control

|  | C. | E. | M. | H. | I. | A. |
| --- | --- | --- | --- | --- | --- | --- |
| C. | 1.00 | 0.08 | -0.01 | 0.04 | 0.06 | 0.02 |
| E. | 0.08 | 1.00 | 0.02 | 0.03 | 0.03 | 0.10 |
| M. | -0.01 | 0.02 | 1.00 | 0.01 | 0.03 | 0.00 |
| H. | 0.04 | 0.03 | 0.01 | 1.00 | 0.08 | 0.03 |
| I. | 0.06 | 0.03 | 0.03 | 0.08 | 1.00 | 0.01 |
| A. | 0.02 | 0.10 | 0.00 | 0.03 | 0.01 | 1.00 |

# Overview of factor score distributions


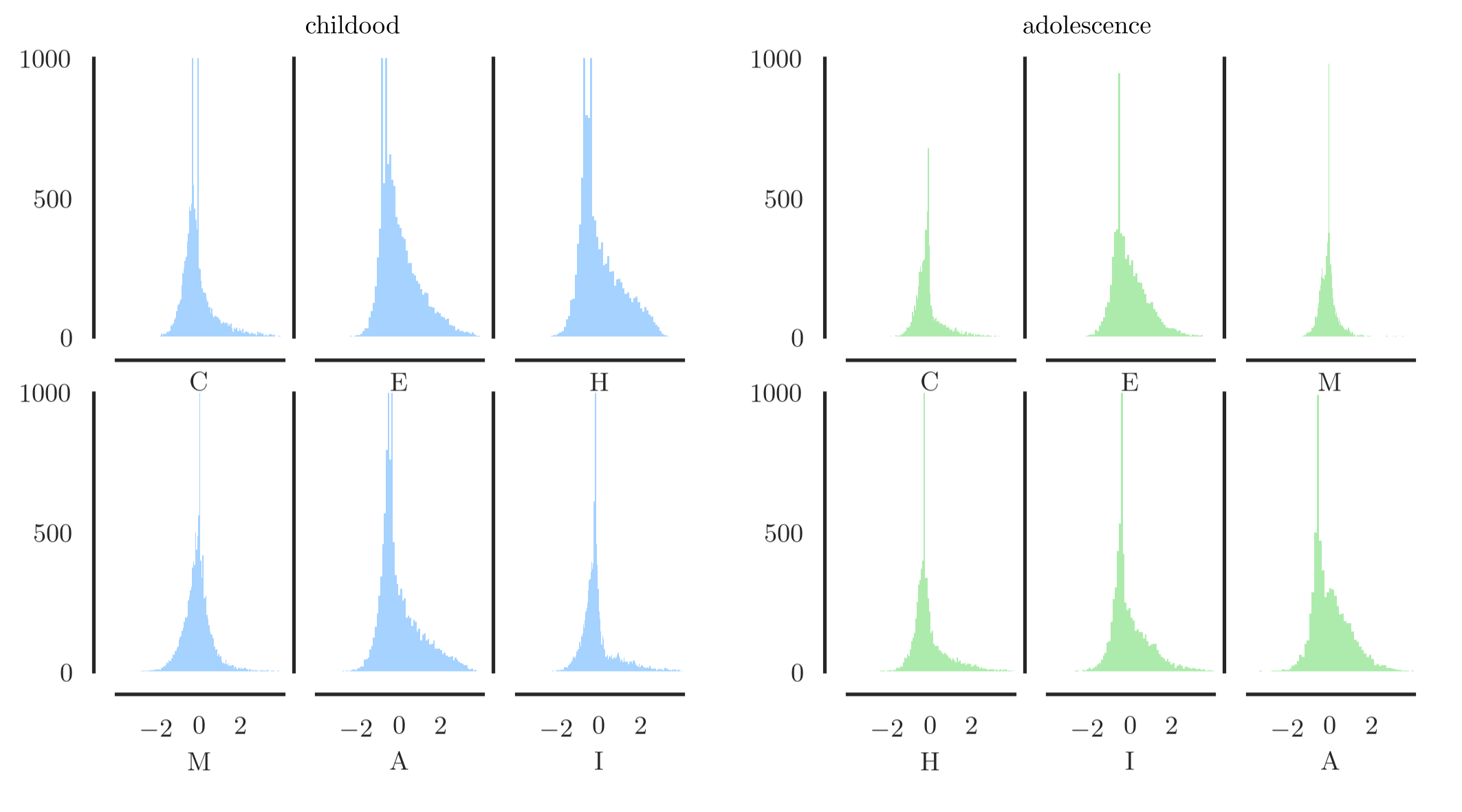


**Figure 4** Overview of the distribution of factor scores for each factor in the childhood (left) and adolescence (right) data. The number of histogram bins was determined using the Freedman-Diaconis estimator.

# Hybrid Hierarchical Clustering

Squared Euclidean distance was used to quantify the similarity between participant’s factor scores. We diverged slightly from the original HHC procedure, because we noticed that the algorithm failed when identical distances were present. The duplicated distances probably occurred because of the large sample size (>6,000) and limited number of indicators (8 factors) in the current analysis. To circumvent this problem, we removed duplicated distances, created the clusters, and associated the left-out entries with the cluster with the shortest distance to them.

# Clustering results using consensus community clustering

The compare the results obtained with HHC, we obtained a clustering solution using consensus community clusters. The methods employed for this analysis were identical to the ones described in Bathelt et al. 2018. Importantly, the similarity between participants was expressed as the Pearson correlation coefficient across factor scores over domains rather than Euclidean distance as in the main analysis. We only included participants that contributed data at the childhood and adolescence assessment for this analysis.

Community clustering of the childhood data indicated three groups (C1: n=1,606 [29.30%], 928 female; C2: 1,843 [33.62%], 1,044 female; C3: n=2,033 [37.09%], 1,107 female). One group did not display any behavioural problems (C3), another group showed problems with Hyperactivity/Impulsivity (C1), and another group showed problems with Emotional Control and Anxiety (C2).

Community clustering of the adolescence data indicated three groups (A1: n=1,821 [27.04%], 987 female; A2: n=2,325 [35.52%], 1,100 female; A3: n=2,589 [38.44%], 1,382 female). One group displayed low ratings of behavioural problems across all domains (A2), one group displayed problems with Emotional Control (A1) and another group showed problems with Anxiety (A3).


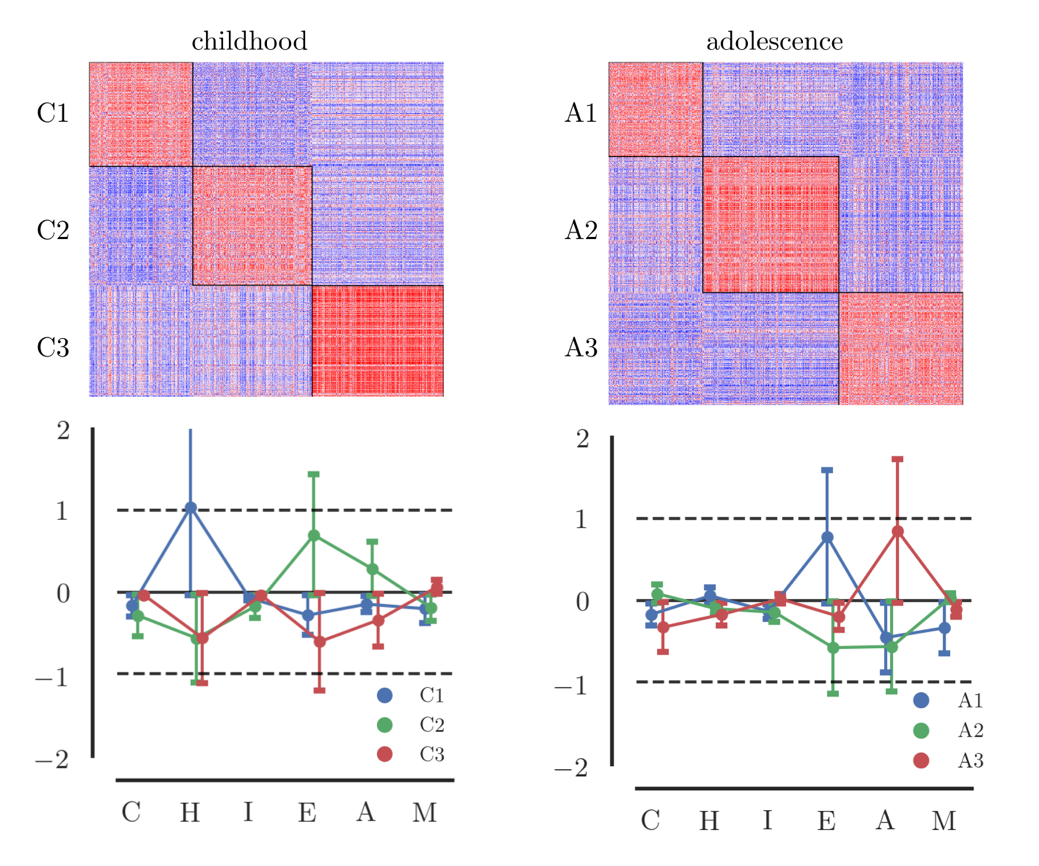


**Figure 5** Results of consensus community clustering. The left panel shows the clustering of childhood data and the right panel shows the results for the adolescence data. The top figures show the participant-by-parent correlation matrix ordered by the consensus clustering solution. The bottom figures show the profiles of factors scores for each clustering-defined group. The values indicate z-scores relative to the entire sample.

# Hybrid hierarchical clustering solution at k=4

**Childhood data**: At the level of 4 clusters, statistical comparison indicated a significant group-by-factor interaction (ANOVA: F(25, 33690)=648.96, *p*<0.001). One group contained children with no behavioural problems across all of the domains (C1, 59.62%, n=7,234). The second group showed problems with Motor Control and Hyperactivity/Impulsivity combined with low Anxiety/Worrying (C2, 12.15%, n=1,474). The third group displayed problems with Anxiety and Motor Control (C3, 10.15%, n=1,231) and the fourth group had problems related to Conduct and Emotional Control (C4, 18.09%, n=2,195).

**Adolescence data:** Using 4 clusters, comparison of the factor scores between the groups indicated a significant group-by-factor interaction (analysis of variance (ANOVA): F(15, 33700)=638.33, *p*<0.001). The first group had low ratings of behavioural problems across all factor scores (A1, 55.67%, n=3,687). The second group displayed problems with Anxiety and Motor Control (A2, 11.08%, n=747). The third group showed more difficulties related to Emotional Control (A3, 25.64%, n=1,729), and the fourth group showed selective Hyperactivity/Impulsivity problems (A4, 8.62%, n=581).

# Descriptive statistics for clustering-defined groups

**Table S7** Descriptive statistics (mean, standard error) for scores in each factor domain at the 4-cluster solution for the childhood data.

|  | C. | | H. | | I. | | E. | | A. | | M. | |
| --- | --- | --- | --- | --- | --- | --- | --- | --- | --- | --- | --- | --- |
|  | mean | SE | mean | SE | mean | SE | mean | SE | mean | SE | mean | SE |
| C1 | 0.84 | 0.025 | 0.21 | 0.026 | -0.12 | 0.013 | 0.60 | 0.027 | 0.14 | 0.025 | -0.77 | 0.012 |
| C2 | -0.21 | 0.006 | -0.45 | 0.006 | -0.23 | 0.004 | -0.33 | 0.008 | -0.19 | 0.009 | 0.01 | 0.004 |
| C3 | -0.43 | 0.018 | 1.08 | 0.023 | -0.65 | 0.015 | 0.37 | 0.025 | 0.28 | 0.029 | 0.53 | 0.017 |
| C4 | -0.25 | 0.019 | 0.35 | 0.032 | 1.67 | 0.026 | 0.04 | 0.029 | 0.20 | 0.03 | -0.60 | 0.016 |

**Table S8** Descriptive statistics (mean, standard error) for scores in each factor domain at the 7-cluster solution for the childhood data.

|  | C. | | H. | | I. | | E. | | A. | | M. | |
| --- | --- | --- | --- | --- | --- | --- | --- | --- | --- | --- | --- | --- |
|  | mean | SE | mean | SE | mean | SE | mean | SE | mean | SE | mean | SE |
| C1a | -0.19 | 0.006 | -0.4 | 0.006 | -0.21 | 0.004 | -0.35 | 0.008 | -0.44 | 0.004 | 0.01 | 0.004 |
| C1b | -0.31 | 0.021 | -0.76 | 0.017 | -0.37 | 0.014 | -0.22 | 0.025 | 1.33 | 0.025 | 0.01 | 0.016 |
| C2a | -0.55 | 0.029 | 1.12 | 0.037 | -0.72 | 0.024 | -0.05 | 0.039 | 1.62 | 0.03 | 0.3 | 0.024 |
| C2b | -0.36 | 0.023 | 1.05 | 0.029 | -0.61 | 0.019 | 0.61 | 0.031 | -0.53 | 0.02 | 0.67 | 0.022 |
| C3 | -0.25 | 0.019 | 0.35 | 0.032 | 1.67 | 0.026 | 0.04 | 0.029 | 0.2 | 0.031 | -0.58 | 0.016 |
| C4a | 0.16 | 0.015 | 0.19 | 0.033 | -0.12 | 0.014 | 0.97 | 0.032 | 0.21 | 0.032 | -0.88 | 0.012 |
| C4b | 1.95 | 0.031 | 0.25 | 0.042 | -0.13 | 0.025 | -0.01 | 0.038 | 0.01 | 0.041 | -0.58 | 0.022 |

**Table S9** Descriptive statistics (mean, standard error) for scores in each factor domain at the 4-cluster solution for the adolescence data.

|  | C. | | H. | | I. | | E. | | A. | | M. | |
| --- | --- | --- | --- | --- | --- | --- | --- | --- | --- | --- | --- | --- |
|  | mean | SE | mean | SE | mean | SE | mean | SE | mean | SE | mean | SE |
| A1 | -0.09 | 0.009 | -0.23 | 0.009 | -0.15 | 0.011 | -0.5 | 0.006 | -0.27 | 0.011 | -0.08 | 0.003 |
| A2 | 0.23 | 0.035 | 0.27 | 0.041 | -0.16 | 0.038 | -0.52 | 0.029 | 0.62 | 0.037 | 0.76 | 0.023 |
| A3 | -0.11 | 0.019 | -0.39 | 0.012 | 0.1 | 0.024 | 0.8 | 0.018 | 0.19 | 0.025 | -0.39 | 0.007 |
| A4 | -0.54 | 0.028 | 1.56 | 0.033 | 0.24 | 0.051 | 0.7 | 0.037 | 0.07 | 0.043 | -0.5 | 0.018 |

**Table S10** Descriptive statistics (mean, standard error) for scores in each factor domain at the 6-cluster solution for the adolescence data.

|  | C. | | H. | | I. | | E. | | A. | | M. | |
| --- | --- | --- | --- | --- | --- | --- | --- | --- | --- | --- | --- | --- |
|  | mean | SE | mean | SE | mean | SE | mean | SE | mean | SE | mean | SE |
| A1a | -0.10 | 0.008 | -0.19 | 0.010 | -0.43 | 0.006 | -0.46 | 0.007 | -0.29 | 0.011 | -0.08 | 0.003 |
| A1b | -0.05 | 0.030 | -0.41 | 0.025 | 1.04 | 0.025 | -0.66 | 0.017 | -0.18 | 0.028 | -0.08 | 0.01 |
| A2 | 0.23 | 0.035 | 0.27 | 0.040 | -0.16 | 0.038 | -0.52 | 0.029 | 0.62 | 0.037 | 0.76 | 0.023 |
| A3a | 0.16 | 0.027 | -0.31 | 0.016 | -0.23 | 0.026 | 0.91 | 0.023 | -0.40 | 0.023 | -0.39 | 0.01 |
| A3b | -0.44 | 0.021 | -0.49 | 0.018 | 0.53 | 0.040 | 0.66 | 0.027 | 0.94 | 0.033 | -0.39 | 0.011 |
| A4 | -0.54 | 0.028 | 1.56 | 0.033 | 0.24 | 0.050 | 0.70 | 0.037 | 0.07 | 0.043 | -0.50 | 0.018 |

# Comparison of bootstrapped Fowlkes–Mallows indices to observed data

­­­­For this analysis, the Fowlkes–Mallows index between the observed childhood data and 1,000 random permutations was calculated to build a bootstrap distribution of random indices. The dendrograms were compared with 4 groups (k=4). Further, to match the cut-off with the observed data, the original dendrogram was cut at k=6 and the bootstrapped dendrograms at k=7. This distribution was compared to the observed adolescence data at k=7 (see Figure S3). The results indicated that the similarity between the clustering based on the childhood and adolescence data were less similar than can be expected by chance.


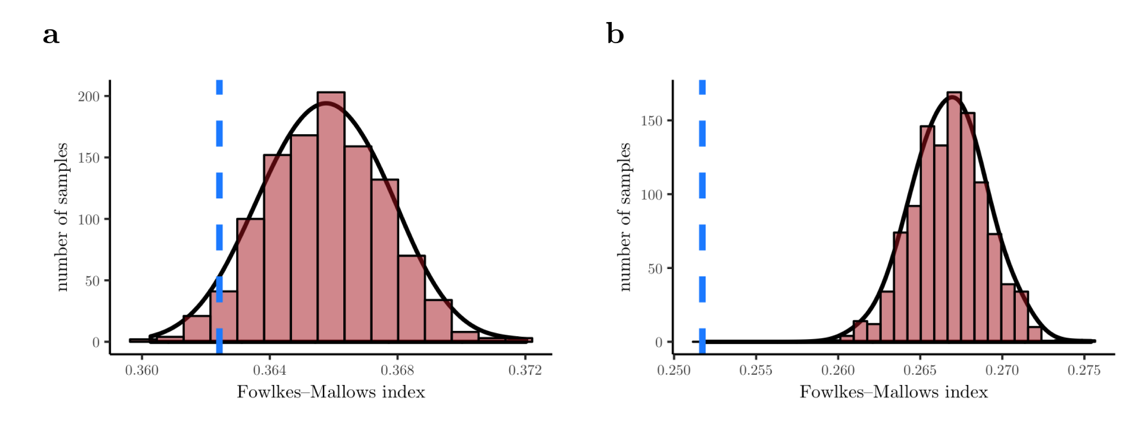


**Figure S6** Results of comparison between observed similarity between the childhood and adolescence data and bootstrapped similarity at k=4 (a) and k=6/7 (b). The distribution of boostrapped Fowlkes–Mallows indices based on random permutation of the childhood data are shown in red. The blue dashed line indicates the observed Fowlkes–Mallows index when comparing the childhood and adolescence dendrograms.

# Overview of demographic, educational, and cognitive variables

**Table 11** Overview of binary demographic variables (childhood data)

|  | Yes |  | No |  | Missing |  |
| --- | --- | --- | --- | --- | --- | --- |
|  | n | % | no | % | n | % |
| immigration background | 618 | 4.16 | 13094 | 88.06 | 1158 | 7.79 |

#
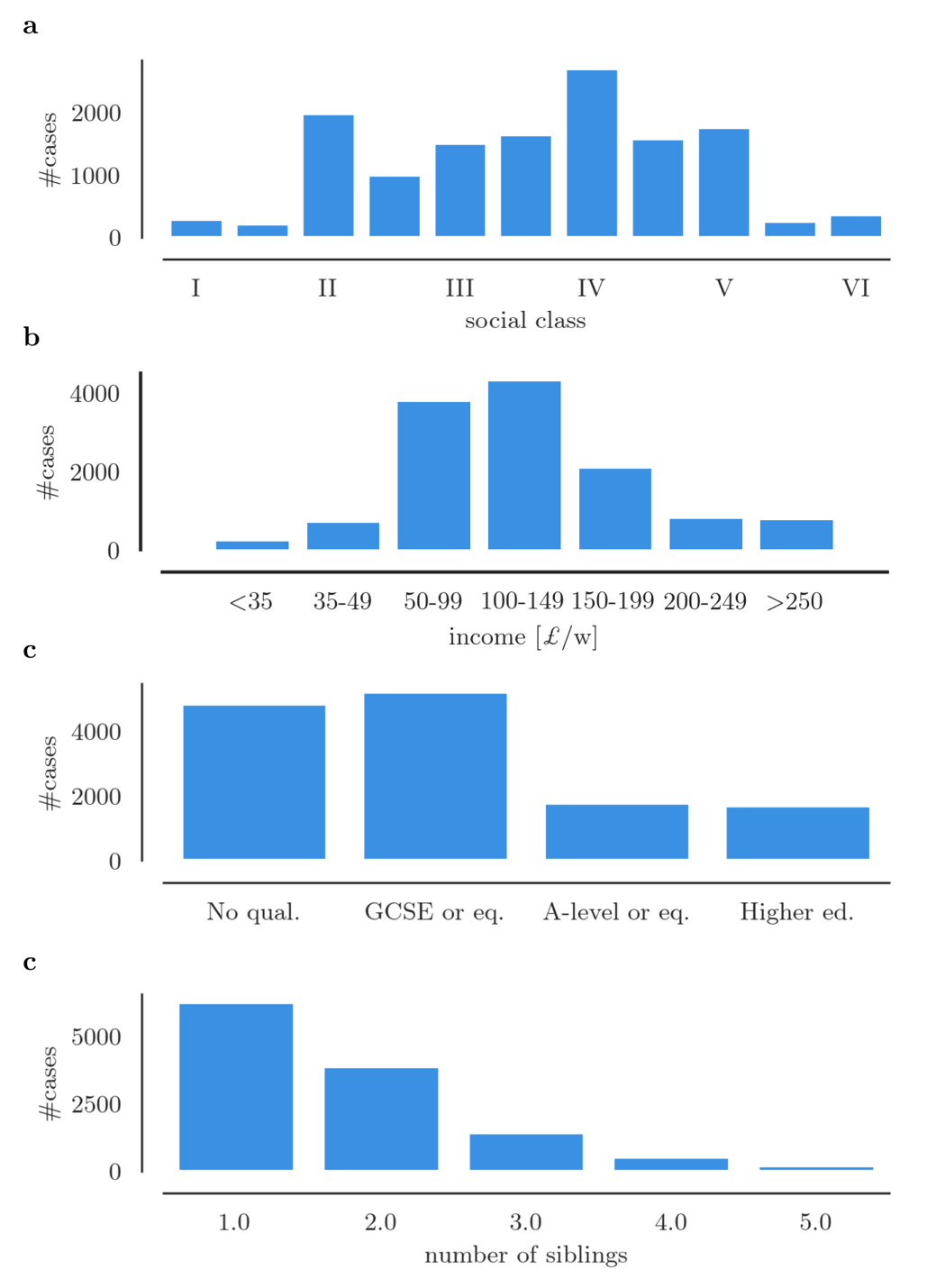


**Figure 7** Overview of the distributions of demographic variables (childhood data). **a** Social class as characterised in the BCS 1980 sweep at age 10 years according to the Hall-Jones scale of occupational prestige (Hall & Jones, 1950). The average across parents was used when data was available for both. Missing responses: n=1,641 (11.04%). **b** Total household income in British pounds per week. Missing responses: n=2,330 (15.67%) **c** Education of most educated parent. No qual.: no professional qualifications., GCSE: General Certificate of Secondary Education, A-level: GCE Advanced level, Higher ed.: Higher education. Missing/excluded responses: n=1,444 (9.71%). **d** Number of siblings. Missing responses: n=1,234 (8.30%).

#
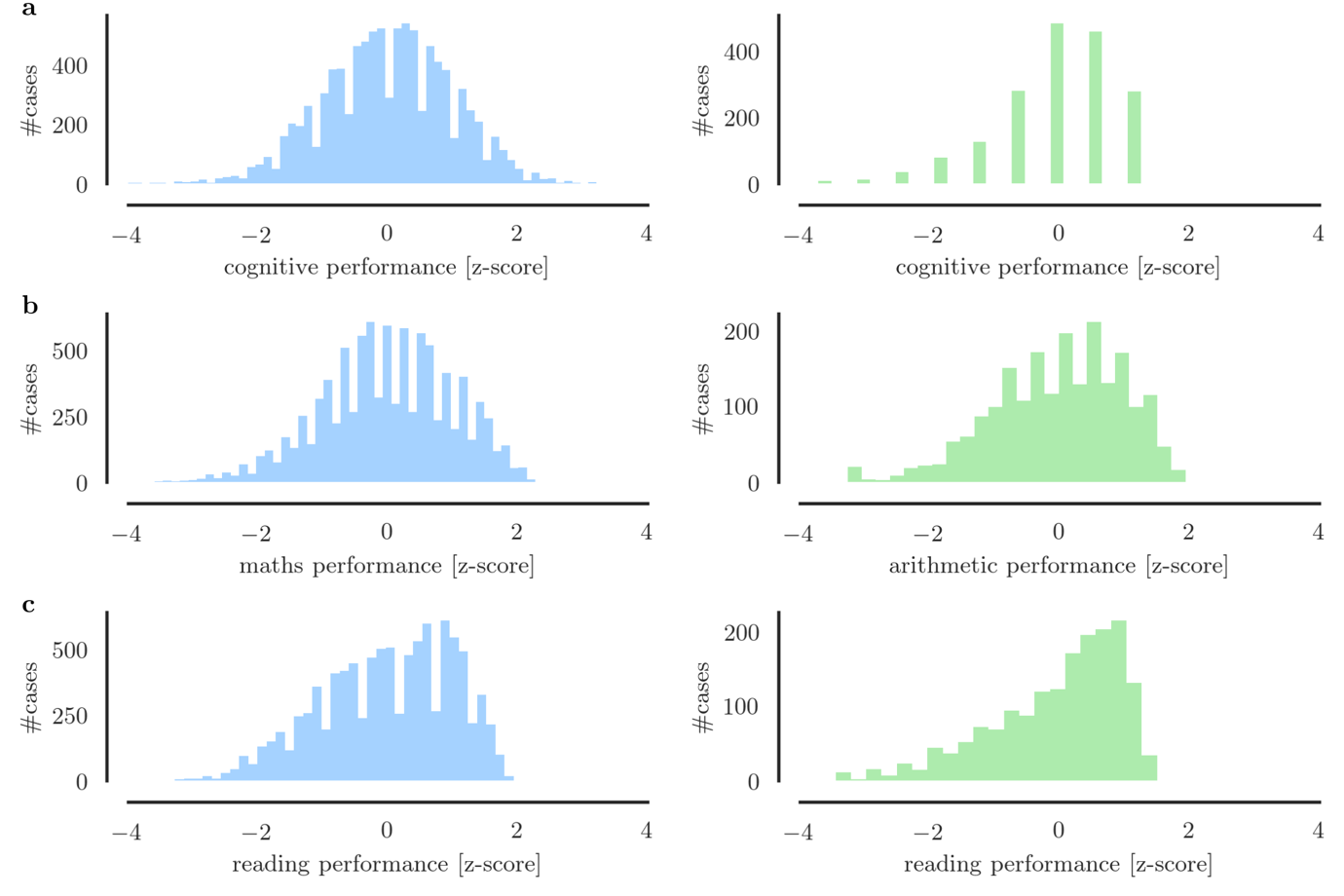


**Figure 8** Distribution of outcome variables. The left side shows the distributions for the childhood data and the right side for the adolescence data. a: cognitive assessments b: maths or arithmetic performance c: reading performance. The number of histogram bins was determined using the Freedman-Diaconis estimator.

# Overview of available and missing data per variable

**Table 12** Overview of missing cases for each variable included in the analysis for the childhood and adolescence data. The percentage indicates the proportion of missing data relative to the number of cases included in the hierarchical clustering analysis. Please note that there was a high number of missing cases for the educational assessments in adolescence because of a teacher strike in that year.

|  | childhood |  | adolescence |  |
| --- | --- | --- | --- | --- |
|  | n | % | n | % |
| cognition | 2394 | 20.55 | 4964 | 73.71 |
| reading | 2550 | 21.02 | 5015 | 74.46 |
| maths | 2555 | 21.06 | 4669 | 69.32 |
| mental health | 648 | 5.34 | 4267 | 63.36 |
|  |  |  |  |  |
| immigration | 1158 | 7.79 | 7 | 0.13 |
| income | 2230 | 15.67 | 414 | 7.55 |
| parental ed. | 1444 | 9.71 | 1138 | 20.76 |
| # siblings | 1234 | 8.30 | 28 | 0.51 |

# Comparison of cognitive, educational, and demographic variables

**Childhood:** There were some demographic differences between the groups. There was a difference between groups in the number of children from single-parent homes (*Χ*^2^=137.03, *p*=0.001). There were more children with an immigration background in C4a (n=53 [7.88%], z=3.72, *p*=0.001), but fewer in C2a (n=26 [2.35%], z=-3.77, *p*=0.001). The groups also showed differences in total family income, the number of siblings, social class, and highest parental education (one-way ANOVA: log-normalized income: F(6,11086)=20.68, *p*=0.001; number of siblings: F(6, 12044)=20.60, *p*<0.001; social-class: F(6, 11708)=43.21, *p*<0.001, highest education: F(6,12127)=22.95, *p*<0.001). Children in C1b, C3, and C4a came from families with a total income below the average across all groups (C1b: t(1126)=-5.15, *p*=0.001; C3: t(687)=-5.74, *p*=0.001; C4a: t(1144)=-4.23, *p*=0.001), lower social class (C1b: t(1177)=8.58, *p*<0.001; C3: t(704)=8.83, *p*<0.001; C4a: t(1192)=3.27, *p*=0.008) and had parents with lower educational qualifications (C1b: t(1187)=-6.65, *p*<0.001; C3: t(728)=-8.03, *p*<0.001; C4a: t(1210)=-3.17, *p*=0.011). Children in C1b and C3 also had a higher number of siblings (C1b: t(1217)=3.05, *p*=0.017; C3: t(732)=9.36, *p*<0.001). Children in C2a came from families with lower education (t(711)=-3.28, *p*=0.008) and lower social class (t(700)=3.44, *p*=0.004). In contrast, children in C1a came from families with higher income (t(5457)=6.34, *p*<0.001), higher education (t(5887)=7.65, *p*<0.001), and higher social class (t(5808)=-8.81, *p*<0.001). In summary, across multiple measures of family structure and socio-economic background, children with anxiety and emotional control problems (C1b, C2a, C3, C4a) appeared less advantaged.

Cognitive, educational, and mental health variables were compared between the clustering defined groups. Comparison of general cognitive performance indicated a main effect of clustering group (n=1,857, one-way ANOVA: F(6, 1850)=15.86, *p*<0.001). Follow-up contrasts indicated better performance for C1a (t=5.26, *p*<0.001, *d*=0.07, Bonferroni critical-*p*=0.008,) and lower performance for C3, and C4b (C3: t=-6.83, *p*<0.001, *d*=-0.25; C4b: t=-3.76, *p*<0.001, *d*=-0.12). The other clustering-defined groups did not differ significantly from the whole sample (*p*>0.1). In short, children with no behavioural problems also had higher cognitive ability than the rest of the sample, whereas children with anxiety or conduct problems had significantly reduced cognitive scores.

The clustering-defined groups also showed differences in academic attainment measures.  Statistical comparison indicated a significant group effect (n=9,579, Reading: F(6, 9577)=89.82, *p*<0.001; Maths: F(6, 9572)=90.30, *p*<0.001; one-way ANOVA). Follow-up analysis indicated a better performance on the reading assessments for C1a (t=14.75, p<0.001, *d*=0.21, Bonferroni critical-*p*=0.007), and worse performance for C1b (t=-5.23, p<0.001, d=-0.17), C2a (t=-9.11, p<0.001, d=-0.38), and C3 (t=-11.23, p<0.001, d=-0.47) and C4b (t=-9.09, *p*<0.001, *d*=-0.33). The other groups fell within the expected range (C2b: t=-0.95, p>0.999, d=-0.03; C4a: t=-1.00, p>0.999, d=-0.03). For performance on the maths assessments, a similar pattern was observed with better performance in C1a (t=14.74, p<0.001, d=0.21), and lower performance in C1b (t=-7.28, p<0.001, d=-0.23), C2a (t=-8.83, p<0.001, d=-0.37), C3 (t=-10.01, p<0.001, d=-0.43), and C4b (t=-9.31, p<0.001, d=-0.33), and no difference for C2b (t=-1.00, p>0.999, d=-0.03) and C4a (t=-0.64, p>0.999, d=-0.02). In short, children with no behavioural problems also tended to have good literacy and numeracy skills. By contrast, children with symptoms of anxiety (C1b), anxiety with hyperactivity (C2a), anxiety with motor problems (C3) and conduct problems (C4b) had significantly poorer educational outcomes.

Regarding mental health, 2.96% (n=340, N=11,486) of the sample were rated as having behavioural or emotional problems on the medical examination form. Some clustering-defined groups contained a higher proportion of these cases than expected by chance (*Χ*^2^=67.19, *p*<0.001) with a higher incidence in C1b (27.79% of all cases, n=97) and C4a (15.76% of all cases, n=55) and a lower incidence in C4b (6.87% of all cases, n=24) and C2a (10.31% of all cases, n=36). Thus, recognised mental health difficulties were more common in children with either anxiety or emotional control symptoms, as rated in the questionnaire.

**Adolescence:** The clustering-defined groups were compared on educational and cognitive attainment measures. Around a third of the sample had matrix reasoning, reading and arithmetic assessment scores available (Matrix Reasoning: 30% n=2,049; Reading: 30%, n=2,049; Arithmetic: 31%, n=2,066). There was no differential data loss between groups, i.e. the frequency of groups in the subsample with cognitive and educational scores did not differ from the frequencies in the whole sample (Matrix Reasoning: *Χ*^2^=1.95, *p*=0.856; Reading: *Χ*^2^=1.95, *p*=0.856; Arithmetic: *Χ*^2^=2.00, *p*=0.849). Statistical comparison indicated no significant group effect for matrix reasoning scores (one-way ANOVA: F(5, 2043)=0.81, *p*=0.545). For the reading assessment, a significant effect of group was found (one-way ANOVA: F(5, 2043)=3.36, *p*=0.005). However, follow-up contrasts did not indicate any deviation from average performance for any group after adjustment for multiple comparisons (one-sample t-test compared to mu=0: all *p*>0.1, Bonferroni critical-*p*=0.008; *d*=-0.13-0.09). For the arithmetic assessment, statistical comparison indicated a significant difference in total arithmetic scores between the clustering-defined groups (one-way ANOVA: F(5, 2060)=15.39, *p*<0.001, Bonferroni critical-*p*=0.008). Higher-than-average scores were observed in A1b (one-sample t-test compared to μ=0: t=4.90, *p*<0.001, *d*=0.14) and lower scores were observed for A3b (t=-6.47, *p*<0.001, *d*=-0.41). Other groups did not deviate significantly from the average (A1a: t=-1.11, *p*>0.999, *d*=-0.07; A2: t=-2.58, *p*=0.066, *d*=-0.06; A3a: t=1.49, *p*=0.822, *d*=0.05; A4: t=-2.47, *p*=0.091, *d*=-0.19).

Mental health ratings on the Medical Assessment Form were available for almost two thirds of the sample (63.31%, n=4.270). Of these adolescents, 1.29% (n=55) were rated as having behavioural or emotional problems. None of the clustering-defined groups contained a disproportionate number of these cases (*Χ*^2^=0.15, *p*>0.999).

# Full reference list of software packages used for the analysis

**R**

Chipman, H. & Tibshirani, R. (2015). *hybridHclust: Hybrid Hierarchical Clustering*. [with *tsvq* code originally from Trevor Hastie]

Fox, J. (2016). *polycor: Polychoric and Polyserial Correlations*.

Galili, T. (2015). dendextend: an R package for visualizing, adjusting, and comparing trees of hierarchical clustering. *Bioinformatics*. http://doi.org/10.1093/bioinformatics/btv428

Karambelkar, B. (2016). *colormap: Color Palettes using Colormaps Node Module*.

Kassambara, A., & Mundt, F. (2017). *factoextra: Extract and Visualize the Results of Multivariate Data Analyses*.

Lai, R. (2018). *arrangements: Fast Generators and Iterators for Permutations, Combinations and Partitions*.

Lawrence, M. A. (2016). *ez: Easy Analysis and Visualization of Factorial Experiments*.

Maechler, M., Rousseeuw, P., Struyf, A., Hubert, M., & Hornik, K. (2018). *cluster: Cluster Analysis Basics and Extensions*.

Nakazawa, M. (2018). *fmsb: Functions for Medical Statistics Book with some Demographic Data*.

Neuwirth, E. (2014). *RColorBrewer: ColorBrewer Palettes*.

Revelle, W. (2017). *psych: Procedures for Psychological, Psychometric, and Personality Research*. Evanston, Illinois.

Sakai, R. (2015). *dendsort: Modular Leaf Ordering Methods for Dendrogram Nodes*.

Wei, T., & Simko, V. (2017). *R package “corrplot”: Visualization of a Correlation Matrix*.

Wickham, H. (2007). Reshaping Data with the reshape Package. *Journal of Statistical Software*, *21*(12), 1–20.

Wickham, H. (2011). The Split-Apply-Combine Strategy for Data Analysis. *Journal of Statistical Software*, *40*(1), 1–29.

Wickham, H. (2016). ggplot2: Elegant Graphics for Data Analysis. Springer-Verlag New York.

Wickham, H., & Henry, L. (2018). *tidyr: Easily Tidy Data with 'spread()' and “gather()” Functions*.

Winston Chang. (2014). *extrafont: Tools for using fonts*.

Xiao, N. (2018). *ggsci: Scientific Journal and Sci-Fi Themed Color Palettes for “ggplot2.”*

**Python**

Hunter, J. D. (2007). Matplotlib: A 2D Graphics. *Environment, Computing in Science & Engineering*, *9*, 90–95. http://doi.org/10.1109/MCSE.2007.55

Jones, E., Oliphant, T., Peterson, P., others. (2001). *SciPy: Open source scientific tools for Python*.

McKinney, W. (2010). Data Structures for Statistical Computing in Python (pp. 51–56). Presented at the Proceedings of the 9th Python in Science Conference.

Oliphant, T. (2006). A guide to NumPy. Trelgol Publishing.

Seabold, S., & Perktold, J. (2010). Statsmodels: Econometric and statistical modeling with python. Presented at the 9th Python in Science Conference.
